# Biosynthesis inhibition of miR-142-5p in a *N*^6^-methyladenosine-dependent manner induces neuropathic pain through CDK5/TRPV1 signaling

**DOI:** 10.1186/s11658-025-00695-w

**Published:** 2025-01-31

**Authors:** Jinshi Li, Yang Guo, Chen Zhu, Dongxu Wang, Yuan Li, Xiaotong Hao, Linyan Cao, Yiting Fan, Bo Fang

**Affiliations:** 1 Department of Anesthesiology, The First Hospital of China Medical University, NO.155, North Nanjing Street, Heping District, Shenyang, 110001 Liaoning China; 2 Department of Surgical Oncology, Breast Surgery, General Surgery, The First Hospital of China Medical University, Shenyang, 110001 Liaoning China; 3 Department of Neurosurgery, The First Hospital of China Medical University, Shenyang, 110001 Liaoning China

**Keywords:** Neuropathic pain, TRPV1, CDK5, *N*^6^-methyladenosine, Cell membrane transport, Phosphorylation

## Abstract

**Background:**

Neuropathic pain (NP) represents a debilitating and refractory condition. However, the understanding of NP and the current treatment approaches available for its management are limited. Therefore, there is a significant need to address the dearth of effective therapeutic interventions. This study aims to investigate the regulation of transient receptor potential vanilloid 1 (TRPV1) and cyclin-dependent kinase 5 (CDK5) expression levels by miR-142-5p as a common upstream molecule, and to delve into the mature process of miR-142-5p from the perspective of *N*^6^-methyladenosine (m^6^A) modification.

**Methods:**

To assess the RNA levels of TRPV1, CDK5, miR-142-5p, pre-miR-142, and pri-miR-142, quantitative PCR with reverse transcription (RT–qPCR) was utilized. Western blot analysis was employed to determine changes in protein expression for TRPV1 and CDK5. For assessing the interaction mechanism and binding site between TRPV1 and CDK5, various techniques were applied, including mass spectrometry, coimmunoprecipitation (co-IP), and glutathione-*S*-transferase (GST)-pulldown assays. The subcellular localization of TRPV1 on the cell membrane was visualized through immunofluorescence, and the translocation was confirmed by western blot analysis after performing membrane-plasma separation in parallel. Moreover, intracellular calcium transport was monitored using calcium imaging as an indicator of cell excitability. The binding of miRNA-142-5p to the 3’UTR of TRPV1 and CDK5 was investigated using the dual-luciferase reporter assay. The overall level of m^6^A was first determined by RNA m^6^A methylation assay, and subsequently the methylation level of pri-miR-142 was assessed using the meRIP assay to detect m^6^A modification. In addition, an in vivo rat chronic constriction injury (CCI) model was established, and miR-142-5p agomir or antagomir was injected intrathecally. An enzyme-linked immunosorbent assay (ELISA) was used to measure the levels of IL-6 and TNF. Paw withdrawal mechanical threshold (PWMT) and paw withdrawal thermal latency (PWTL) were examined.

**Results:**

The expression levels of TRPV1 and CDK5 were found to be upregulated not only in the in vivo CCI model but also in the in vitro lipopolysaccharide (LPS) treatment cell model as well. CDK5 was observed to phosphorylate TRPV1 at T406, prompting the translocation of TRPV1 to the cell membrane and consequent augmentation of cellular excitability. Notably, CDK5 was found to directly bind to TRPV1, and the binding region was localized within the 1–390 amino acid sequence of TRPV1. According to database predictions, miR-142-5p, identified as a shared upstream molecule of TRPV1 and CDK5, exhibited downregulation following induction by NP. MiR-142-5p was shown to simultaneously bind to the mRNA of CDK5 and TRPV1, thereby inhibiting their expression. After LPS treatment, it was observed that pri-miR-142 expression increased, while pre-miR-142 and miR-142-5p expression decreased, suggesting inhibition of the maturation process of pri-miR-142. In addition, the overall level of m^6^A and in particular the pri-miR-142 m^6^A modification increased upon LPS treatment. Knockdown of METTL14 led to decreased pri-miR-124 expression, increased pre-miR-124 expression, and enhanced mature miR-142-5p expression, indicating the relief of miR-142-5p maturation repression. The in vivo results indicated that miR-142-5p negatively regulated the expression of CDK5 and TRPV1, suppressed the expression of inflammatory factors IL-6 and TNF, and improved the PWMT and PWTL.

**Conclusions:**

In this study, we perform a thorough investigation to examine the effects of CDK5 and TRPV1 on NP, elucidating their binding relationship and the impact of CDK5 on the membrane transport of TRPV1. Notably, our findings reveal that miR-142-5p, acting as a crucial upstream molecule, exhibits inhibitory effects on the expression of both CDK5 and TRPV1. Moreover, we observe that METTL14 facilitates the m^6^A modification of pri-miR-142, thereby impeding the maturation transition of pri-miR-142 and ultimately leading to the downregulation of mature miR-142-5p.

**Graphical Abstract:**

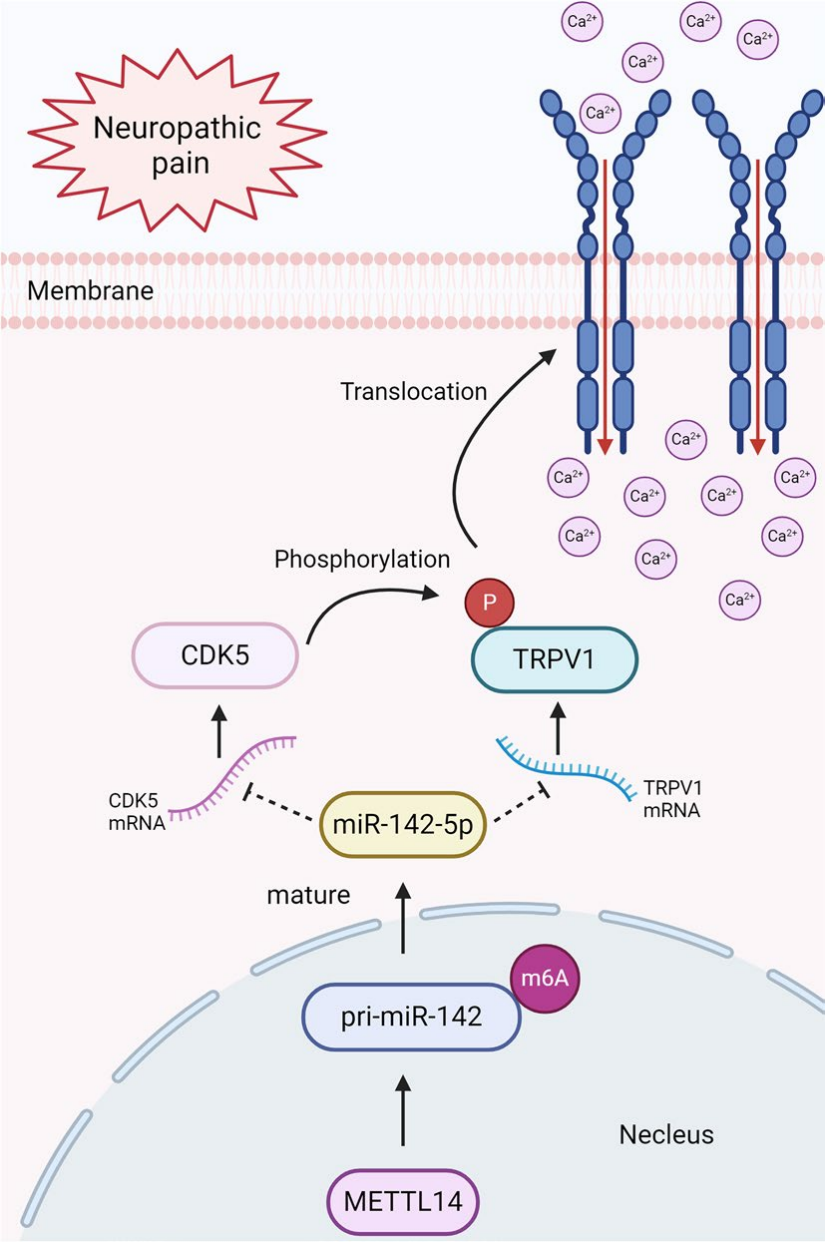

**Supplementary Information:**

The online version contains Supplementary Material available at 10.1186/s11658-025-00695-w.

## Background

In recent years, neuropathic pain (NP) has gained increasing attention within the medical community. Following the successful treatment of certain diseases, a subset of patients may experience ongoing and debilitating chronic pain, such as in cases of shingles, stroke, and severe trauma. It is estimated that approximately one in six individuals is affected by chronic pain, and the profound adverse impact of incessant pain on patients’ lives should not be underestimated [1, 2]. Abnormal pain and hyperalgesia represent prominent symptoms observed in nearly 15–50% of patients with NP, and these manifestations are common in various peripheral neuropathies and central pain disorders [3]. Dorsal root ganglion (DRG) contains the cell bodies of primary sensory neurons, some of which are nociceptive neurons. After experiencing a painful injury, maladaptive molecular changes occur in the cell bodies and axons of these primary sensory neurons within the DRG. Such changes contribute to the development of hypersensitivity and overexcitation of the sensory neurons, a phenomenon known as peripheral sensitization, which plays a pivotal role in the initiation and perpetuation of chronic pain [4, 5].

The transient receptor potential (TRP) channels are a crucial group of receptors responsible for pain signaling in DRG neurons. These channels are composed of four subunits and function as nonselective cation channels. Among the 28 TRP channel isoforms found in mammals, almost all of them conduct calcium ions [5–7]. Transient receptor potential vanilloid 1 (TRPV1), an important member of the TRP channel family, is preferentially expressed in sensory neurons of the peripheral nervous system. It is predominantly found in small and medium nociceptor neurons of DRG, trigeminal ganglion, and sympathetic ganglion [8–10]. The modulation of pain signaling involves the alteration of protein phosphorylation mediated by protein kinases. Cyclin-dependent kinase 5 (CDK5), one such kinase, exhibits significant epigenetic regulation during NP. Its upregulation contributes to the pathogenesis of chronic pain [11]. CDK5 and its activator, p35/p25, are abundantly expressed in spinal nerves, DRG, spinal dorsal horn, and trigeminal ganglion [12–14]. Previous studies have demonstrated that CDK5-mediated phosphorylation of TRPV1 at T407 (in humans and mice) and T406 (in rats) facilitates receptor transport to the plasma membrane and regulates Ca^2+^ influx [15]. However, the detailed interaction mechanism between these molecules and the development of novel drugs in this context remain poorly investigated. Hence, one purpose of this study is to investigate the binding mechanism and explore common upstream regulatory molecules involved in this process.

A growing emphasis on microRNAs (miRNAs) has taken place in recent years owing to their role as noncoding RNA molecules capable of regulating protein expression [16]. MiRNAs have demonstrated potential in regulating the expression of specific genes relevant to the central nervous system and have implications in the development of chronic pain in the peripheral nervous system [11, 17, 18]. Consequently, interfering with miRNA expression in the primary afferent pathway has shown to be effective in inhibiting the long-term development of injury-induced chronic pain-related behaviors [19]. The biosynthesis of miRNA is affected by many factors, notably *N*^6^-methyladenosine (m^6^A) modification plays an important role in regulating this process [20, 21]. In this study, we focus on three aspects: the binding and direct action of TRPV1 and CDK5, the regulatory mechanism of upstream miRNA on CDK5 and TRPV1, and the effect of m^6^A modification on miRNA biosynthesis.

## Methods

### Animal

Our primary objective is to explore a novel mechanism and potential therapeutic target involving the progression of NP. Sample size was considered to empirically estimate variation in results and the required statistical power while minimizing the number of animals. Seven-week-old male Sprague-Dawley (SD) rats were randomly assigned to each group, with five rats per group for each experiment to ensure the reproducibility of the observed results. The study was not blinded. Animal experiments in this study were conducted in accordance with the Guide for the Care and Use of Laboratory Animals published by the US National Institutes of Health. All procedures were reviewed and approved by the Ethics Committee of the Animal Department of China Medical University (KT2023866).

### Chronic constriction injury (CCI) surgery and behavioral analysis

The CCI model of rats was established following established protocols [22]. Under intraperitoneal anesthesia using sodium pentobarbital (50 mg/kg), the rats were positioned in the ventral decubitus position following stabilization of anesthesia. A lateral incision was made slightly lateral to the right iliac crest, perpendicular to the hind limb, allowing for blunt dissection to locate the sciatic nerve. A section of approximately 7 mm in length was isolated along the sciatic nerve and then ligated using a 4–0 gut cord with 1 mm spacing in front of the three branches. Attention was paid to ensure adequate knot tightness, as confirmed by the slight twitching of the toe tip and sliding of the knot over the nerve. Following irrigation with normal saline, the local area was sutured intermittently, encompassing the muscle, subcutaneous tissue, and skin. Postoperatively, the rats were housed in a warm and comfortable environment to facilitate recovery. The sham group underwent identical surgical procedures as the model group, with the exception of sciatic nerve ligation.

The paw withdrawal mechanical threshold (PWMT) was assessed following the method described by Bennett [22]. Static allodynia in rats was evaluated at multiple time points (1, 3, 5, 7, and 14 days) post CCI surgery. To acclimate to the testing environment, all rats underwent an adaptation period of approximately 15 min in a ventilated plastic box with a wire mesh bottom. Static allodynia was then assessed by applying von Frey hairs (Danmic Global, American) to the plantar surface of the hind paw on the affected side, increasing the force until a withdrawal response was observed. Subsequently, the minimum force required to induce the paw withdrawal response was recorded as the PWMT. To evaluate thermal hypersensitivity to heat stimulus in a rat model, the hot plate analgesia meter (Yuyanbio, Shanghai, China) was employed with slight modifications to the previously described methods [22]. To assess thermal hypersensitivity, rats were individually placed on a hot plate set to a temperature of 50.5 °C. The latency period, defined as the time taken for each rat to either lick its hind paw or show a jumping response, was measured and recorded.

### Intrathecal injection

In accordance with previously published methodologies [18, 23], the hair around the waist of the rats was removed, and the skin was sterilized using iodophor. Lumbar puncture was conducted by gently grasping the iliac crest of the rats and vertically inserting a 10-μl Hamilton microsyringe with a 30-gauge needle into the intervertebral space between L4 and L5. Successful puncture was confirmed by observing a tail shiver or flick. The liquid was injected evenly and slowly over approximately 15 s. As described in the study conducted by Zhang et al., the miR-142-5p agomir (Huzhou Hippo Biotechnology Co, LTD. Huzhou, China) 1 nmol/day was injected prior to CCI surgery and administered continuously for 5 days [24]. Following 5 days of continuous injection of antagomir (Huzhou Hippo Biotechnology Co, LTD. Huzhou, China) 1 nmol/day, subsequent experiments were conducted. The sequence information for agomir was: CAUAAAGUAGAAAGCACUACU; The sequence information for antagomir was: AGUAGUGCUUUCUACUUUAUG.

### Cell culture and transfection

The DRG cell line used in this study was obtained from the Beijing Beina Chuanglian Biotechnology Institute. The cells were maintained in complete medium composed of Dulbecco’s modified Eagle medium (DMEM) supplemented with 10% fetal bovine serum (FBS) under standard conditions of 37 °C, 5% CO_2,_ and a water-saturated atmosphere. Upon reaching 80–100% confluency, the cells were detached using 0.25% trypsin and evenly distributed into three 25 cm^2^ culture flasks. Subsequently, when the cell density reached 75–85%, a mixture of Lipo3.0 (Hanbio Biotechnology Co, LTD. Shanghai, China) and plasmid (Syngenbio Co, LTD. Shanghai, China) was added to serum-free DMEM culture for a duration of 10 min; subsequently, the cells were cultured with the prepared mixture. After 12 h of incubation, the medium was replaced with serum-containing medium, and the cells were further cultured for 24 h.

The 293T cell line was maintained in a complete medium consisting of DMEM supplemented with 10% fetal bovine serum (FBS) under standard conditions of 37 °C, 5% CO_2_ and a water-saturated atmosphere. Given that 293T cells have relatively low adhesion ability, Corning CellBIND (3289) culture flasks are used. The plasmid transfection process of 293T cells was identical to that of the aforementioned DRG cells. The list of plasmids is provided in Supplementary Table 6.

For the transfection of mimics and inhibitors in cells, InvitroRNA™ transfection reagent (InvivoGene Biotechnology, Nanjing, China) was used. When the cell density reached 80–85%, the in vitro transfection reagent was mixed with the mimics or inhibitors and added to the culture medium containing serum. After incubation for 12 h, the cell state was observed and the culture medium was changed in time, followed by further culture for 24 h.

### Cell membrane/cytoplasmic separation

The membrane and cytoplasm of the cells were separated using a plasma membrane protein separation detection kit (Beijing Solarbio Science and Technology Co, Ltd. Beijing, China). Cold protein extract A and C were supplemented with protease inhibitor (1:250) and kept on ice for subsequent use. A total of 5–10 × 10^6^ cells were collected, thoroughly washed to remove pancreatic enzymes and culture medium, and then mixed with 200–400 μl of liquid A. The mixture was vigorously shaken for 20–30 min until complete cell lysis was observed and significant reduction in cell precipitation was achieved. The resulting lysate was centrifuged at 4 °C (12,000*g*) for 5 min to eliminate the supernatant, followed by incubation in a water bath at 37 °C for 5–10 min. Subsequently, centrifugation at 37 °C (100*g*) for 5 min allowed for the separation of the lower layer, which contained the cell membrane, and the upper layer, which contained the cytoplasm, among others. The cell membrane was purified using repeated centrifugation with B reagent. Finally, the cell membrane was fully dissolved with liquid C in preparation for subsequent experiments.

### Calcium imaging

DRG neurons were cultured in a standardized manner in 24-well plates. In the 24-well plate with a bottom area of 2 cm^2^, cells were counted using a cell counting chamber, and approximately 10,000 cells were planted in each well for calcium imaging experiments. Upon reaching approximately 70% confluency, the cells were exposed to a working solution of Fluo-4, AM (Beijing Solarbio Science and Technology Co, Ltd. Beijing, China). The culture was maintained at 37 °C for 20 min. Subsequently, the cells were incubated in Hank’s balanced salt solution (HBSS) supplemented with 1% FBS at a volume equivalent to five times the original volume for 40 min. Afterward, the cells were rinsed thrice with HEPES buffer saline and cultured for an additional 10 min. Next, capsaicin was used to evoke the calcium transients before calcium imaging. The cells were observed for fluorescent Ca^2+^ levels using a fluorescein isothiocyanate (FITC) filter.

### Western blot analysis

In the protein extraction process, a working solution of phenylmethylsulfonyl fluorid (PMSF) in a ratio of 1:50 was prepared with radioimmunoprecipitation assay (RIPA) buffer. This solution was subsequently added to cell or tissue samples for ultrasonic crushing, taking precautions to prevent excessive heat generation that could lead to protein degradation. During the separation of TRPV1 and CDK5 proteins, 10% polyacrylamide gels were employed in the polyacrylamide gel electrophoresis procedure. TRPV1 proteins were transferred to a polyvinylidene fluoride (PVDF) using a wet stain transfer system, applying 250 mA for 90 min. For CDK5, transfer was carried out at 150 mA for 60 min. The PVDF membranes were blocked using 5% skim milk in triple buffer supplemented with 0.05% Tween 20 (TBST) for a duration of 2 h. The primary antibody was incubated overnight at 4 °C, followed by three washes with TBST (each lasting 5 min) and subsequent incubation with the secondary antibody (1:10,000, Abmart, Shanghai, China) at room temperature for 1 h, then washed with TBST three times. The signal was detected using an enhanced chemiluminescence (Tanon, Shanghai, China). The list of antibodies is available in the Supplementary Materials. The list of antibodies is provided in Supplementary Table 5.

### Real-time quantitative reverse transcription PCR (RT–qPCR)

The cell culture dish was washed twice with PBS, followed by the addition of 1 ml TRIzol (Takara, Japan), which was then blown and mixed to ensure full cracking. Subsequently, the sample was collected. To the sample, 200 μl chloroform was added, followed by vigorous shaking and mixing for 30 s to promote thorough mixing, and the mixture was left to stand for 3–5 min at room temperature. After the solution became clearly stratified, it was centrifuged at 4 °C (12,000*r*) for 15 min. The colorless upper transparent liquid was gently pipetted into a fresh Eppendorf tube and thoroughly mixed after adding an equal volume of isopropyl alchol. The mixture was then centrifuged again at 4 °C (12,000*r*) for 15 min. The resulting RNA precipitation was collected, and subjected to two washes with 75% ethanol before being dried. MirRNA and mRNA were extracted together, and the cDNA was obtained by reverse transcription using A-tail method.

Subsequently, the concentration of total RNA was measured by a NanoDrop Microvolume Spectrophotometer (Thermo Fisher Scientific, Massachusetts, USA) and reverse transcription and amplification were carried out (Takara, Japan). The miRNA molecules have a relatively short sequence. Hence, in this experiment, reverse transcription was carried out using the A-tailing approach to obtain cDNA. Finally, threshold cycle (Ct) values were determined using the SYBR dye method (Takara, Japan). *GAPDH* and *U6* were used as housekeeping genes for control. The primers are displayed in Supplementary Table 4.

### Immunofluorescence

The cells were cultured in 24-well plates until complete attachment to the substrate. Following euthanasia of rats using excess sevoflurane, the DRG ganglion was extracted and embedded in paraffin. Paraffin sections were subjected to dewaxing and antigen retrieval. Subsequently, both cell and tissue specimens were fixed, cell membranes were permeabilized, and nonspecific binding sites were blocked. Cell specimens were incubated overnight with primary antibody (1:200, Abmart, Shanghai, China), followed by a 1-h incubation with secondary antibody (1:100, Abmart, Shanghai, China). DAPI staining was performed for 5 min prior to observation under an EVOS FL Auto 2 (Thermo Fisher Scientific, USA). Tissue specimens were incubated overnight at 4 °C with calcitonin gene-related peptide (CGRP), isolectin-B4 (IB4), neurofilament 200 (NF200), and TRPV1, followed by a 2-h incubation with a fluorescent secondary antibody at room temperature. DAPI (Servicebio Technology Co. LTD., Wuhan, China) staining was performed for 5 min, and microscopic examination was carried out thereafter.

### Mass spectrometry

The gels sample stained with Coomassie blue had the band of interest excised and split evenly about 1 mm^3^ for every gel slice. The whole peptide sample was resuspended with 0.1% TFA and desalted with the C18 Cartridge, after which it was resuspended with 10 μl 0.1% formic acid. The samples were separated by chromatography and then mass spectrometry was performed by the Q Exactive Plus mass spectrometer.

### Coimmunoprecipitation (co-IP)

Cell suspensions in RIPA lysate were subjected to centrifugation at 4 °C (12,000*r*) for 15 min to remove cellular debris. The antibody solution (dissolved in binding buffer) was mixed with magnetic beads (Selleckchem, Houston, TX, USA) and thoroughly combined on a rotary mixer for 8–12 h. The magnetic bead-antibody complex was then isolated using a magnetic frame and incubated with the protein lysate for 8–12 h. Following isolation of the magnetic-globin-antibody complex, 30 μl of loading buffer was added to the magnetic bead, and the mixture was subjected to heating at 95 °C for 5 min. Subsequently, a western blot assay was conducted to assess protein binding and confirm molecular interactions.

### GST pulldown

The recombinant plasmid, consisting of the target protein and glutathione-*S*-transferase (GST) fusion tag, was introduced into the DE3 (Beijing Beina Chuanglian Biotechnology Institute) strain. The transformed strains were selected and maintained using ampicillin. For amplification of the transformed strains, a Luria–Bertani (LB) medium was employed, and protein synthesis was induced by the addition of isopropyl β-d-1-thiogalactopyranoside (IPTG). Subsequently, the bacteria were harvested through centrifugation, and protein samples were extracted via ultrasonic fragmentation. The samples were purified using GST magnetic beads (Beijing Solarbio Science and Technology Co, Ltd. Beijing, China) and then combined in vitro with protein solutions derived from cells. Finally, the protein bound to the eluted magnetic beads was assessed through SDS–PAGE and western blot techniques.

### Dual-luciferase reporter assays

Dual-luciferase reporter gene plasmids were designed on the basis of the predicted results obtained from Targetscan and miRWalk databases, found at Reputation Biology. The vector plasmid and Lipo3.0 (Hanbio Biotechnology Co, LTD. Shanghai, China) were mixed in a predetermined ratio and added to a serum-free medium for thorough mixing. The resulting mixture was used to transfect 293T cells, which were subsequently incubated together. After 12–16 h of incubation, the cells were cultured for an additional 24 h with fresh medium. Upon completion of the cell preparations, they were split and subjected to centrifugation to obtain the supernatant. The supernatant was then transferred to a 96-well plate. Firefly detection solution and sea kidney detection solution (Beyotime Biotech Inc, Shanghai, China) were introduced into each well, and the resulting fluorescence intensity was measured using the chemiluminescence method.

### RNA m^6^A quantification

Total RNA was extracted using TRIzol reagent (Takara, Japan). The m^6^A content in total RNA was evaluated using an m^6^A RNA Methylation Quantification Kit (Colorimetric) (Epigentek Group Inc., Farmingdale, NY, USA) according to the manufacturer’s instructions, as reported previously [25]. Equal amounts of total RNA (200 ng) were bound to strip wells using an RNA high binding solution. The m^6^A was captured and detected using the specific capture antibody and detection antibody. Then, the detected m^6^A signal was enhanced using an enhancer solution and quantified colorimetrically after adding color developing solutions by reading the absorbance at a wavelength of 450 nm in a microplate spectrophotometer.

### Methylated RNA immunoprecipitation (MeRIP)

The MeRIP procedure was conducted following the guidelines provided by the manufacturer, using MeRIP™ m^6^A kit (Guangzhou Epibiotek Co, Ltd. Guangzhou, China). Briefly, purified mRNA underwent DNase I treatment for the purpose of removing DNA contaminants, followed by fragmentation using RNA fragmentation reagents. The fragmented RNA was then precipitated using glycogen and sodium acetate. The resulting RNA fragments were subjected to an overnight incubation at 4 °C on a rotator with the antibody-magnetic bead conjugate for immunoprecipitation. Subsequently, the immunoprecipitated RNA was purified and washed multiple times with a wash buffer to isolate the target RNA. Following reverse transcription of the aforementioned RNA, RT–qPCR was performed for quantitative analysis.

### Enzyme-linked immunosorbent assay (ELISA)

The ELISA procedure was conducted in accordance with the manufacturer’s instructions, using the enzyme-linked immunosorbent assay kit (BOSTER Biological Technology Co, LTD. Wuhan, China). Briefly, 100 μl of diluted standard and diluted sample were added to separate wells of a 96-well plate and incubated for 90 min at room temperature. Following blotting of the reaction solution, 100 μl of biotin-labeled antibody (1:100) was added and incubated for 60 min. Subsequently, the wells were washed three times, and 100 μl of peroxidase-labeled avidin (1:100) was added and incubated for 30 min. After five additional washes,90 μl of 3,3′,5,5′-tetramethylbenzidine (TMB) was added to each well and incubated in the dark at 37 °C for 20 min. Finally, the reaction was terminated by adding 100 μl of termination solution, and a microplate reader was used to measure the absorbance at 450 nm.

### Fluorescence in situ hybridization (FISH) assay

After extracting from the sham group and CCI group, the DRG tissues were embedded in paraffin and then sectioned into thin slices with a thickness of approximately 4 μm. The miRNA-142-5p probe was designed and synthesized by GenePharma, and the fluorescence detection was performed using the FISH reagent kit (GenePharma, Shanghai, China). Observe and image using the EVOS FL Auto 2 (Thermo Fisher Scientific, USA). The slide was imaged using the EVOS FL Auto 2 (Thermo Fisher Scientific, USA).

### Statistical analysis

The data were presented as mean ± standard error of the mean (SEM). Statistical analyses were conducted using GraphPad Prism version 9.5.0 (GraphPad Software, USA) and IBM SPSS version 26 (IBM Inc., Armonk, USA). The *t*-test was utilized to compare data between two groups, while one-way analysis of variance (ANOVA) was employed to assess statistical differences between groups in cases involving multiple groups. Repeated-measures analysis of variance was used to compare the differences between the two groups at different time points. Statistically significant was considered when *P*-value < 0.05.

## Results

### CCI upregulates the expression of CDK5 and TRPV1 in the rat DRG

**Fig. 1:**
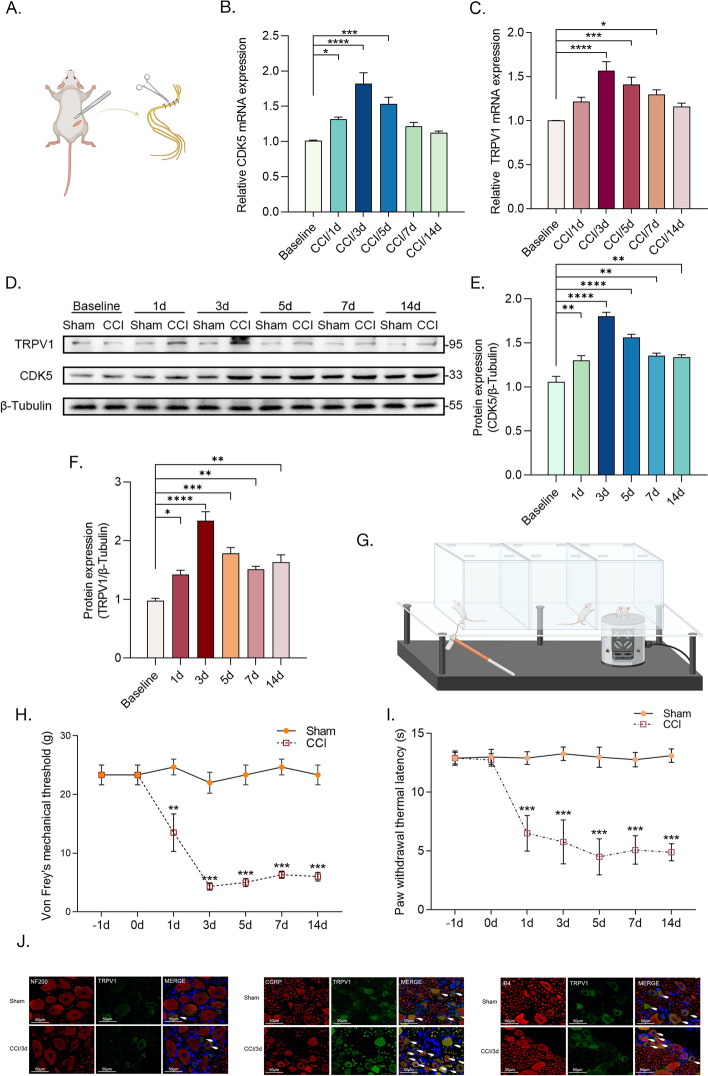
CCI upregulates the expression of transient receptor potential vanilloid 1(TRPV1) and cyclin-dependent kinase 5 (CDK5) in the rat dorsal root ganglion (DRG). **A** Schematic diagram of the operation for chronic constriction injury (CCI) model establishment. The figure was created with Biorender.com. **B** and **C** The relative mRNA expression levels of CDK5 (**B**) and TRPV1 (**C**) in DRG at 1–14 days after CCI (*n* = 5 per group). **D** Western blot results showed the protein expression of TRPV1 and CDK5. **E** and **F** The relative protein expression levels of CDK5 (**E**) and TRPV1 (**F**) in DRG 1–14 d after CCI (*n *= 5 per group). **G** Schematic representation of the paw withdrawal mechanical threshold (PWMT) and paw withdrawal thermal latency (PWTL) measurements. The figure was created with Biorender.com. **H** and **I** PWMT (**H**) and PWTL (**I**) in rats 1–14 days after CCI (*n *= 6 per group). **J** Staining of TRPV1 (green fluorescence), neurofilament 200 (NF200, red fluorescence), calcitonin gene-related peptide (CGRP, red fluorescence), isolectin-B4 (IB4, red fluorescence), and DAPI (blue fluorescence) in sham and CCI group DRG tissue (scale bar, 50 μm). *p < 0.05; **p < 0.01; ***p < 0.0005; ****p < 0.0001

The rat CCI model was established to induce NP, and the DRGs at the L4–6 spinal levels were collected at specific time points (1, 3, 5, 7, and 14 days) following the surgery (Fig. 1A). The RT–qPCR results revealed a significant increase in CDK5 and TRPV1 mRNA levels, reaching a peak on the third day after surgery (Fig. 1B and C). Moreover, western blot experiments were conducted to evaluate the protein levels, demonstrating a consistent upregulation of both CDK5 and TRPV1, peaking on the third day after the establishment of CCI (Fig. 1D–F). Finally, we conducted assessments of mechanical pain threshold and thermal pain threshold in rats at various time points (Fig. 1G). The findings demonstrated a significant decrease in both PWMT and paw withdrawal thermal latency (PWTL) immediately following ligation, reaching the lowest point on the third or fifth day; however, the thresholds did not return to normal levels by the 14th day (Fig. 1H and I). TRPV1 was expressed in NF200-positive, CGRP-positive, and IB4-positive neurons. On the third day of CCI modeling, the expressions of TRPV1 in CGRP- and IB4-positive neurons were conspicuously elevated. By contrast, the increase in its expression in NF200-positive neurons was relatively minor. (Fig. 1J). These findings suggest a potential correlation between alterations in CDK5 and TRPV1 protein expression levels within DRG and changes in pain perception subsequent to CCI.

### Lipopolysaccharide (LPS) upregulates the expression of CDK5 and TRPV1 in DRG neurons

**Fig. 2:**
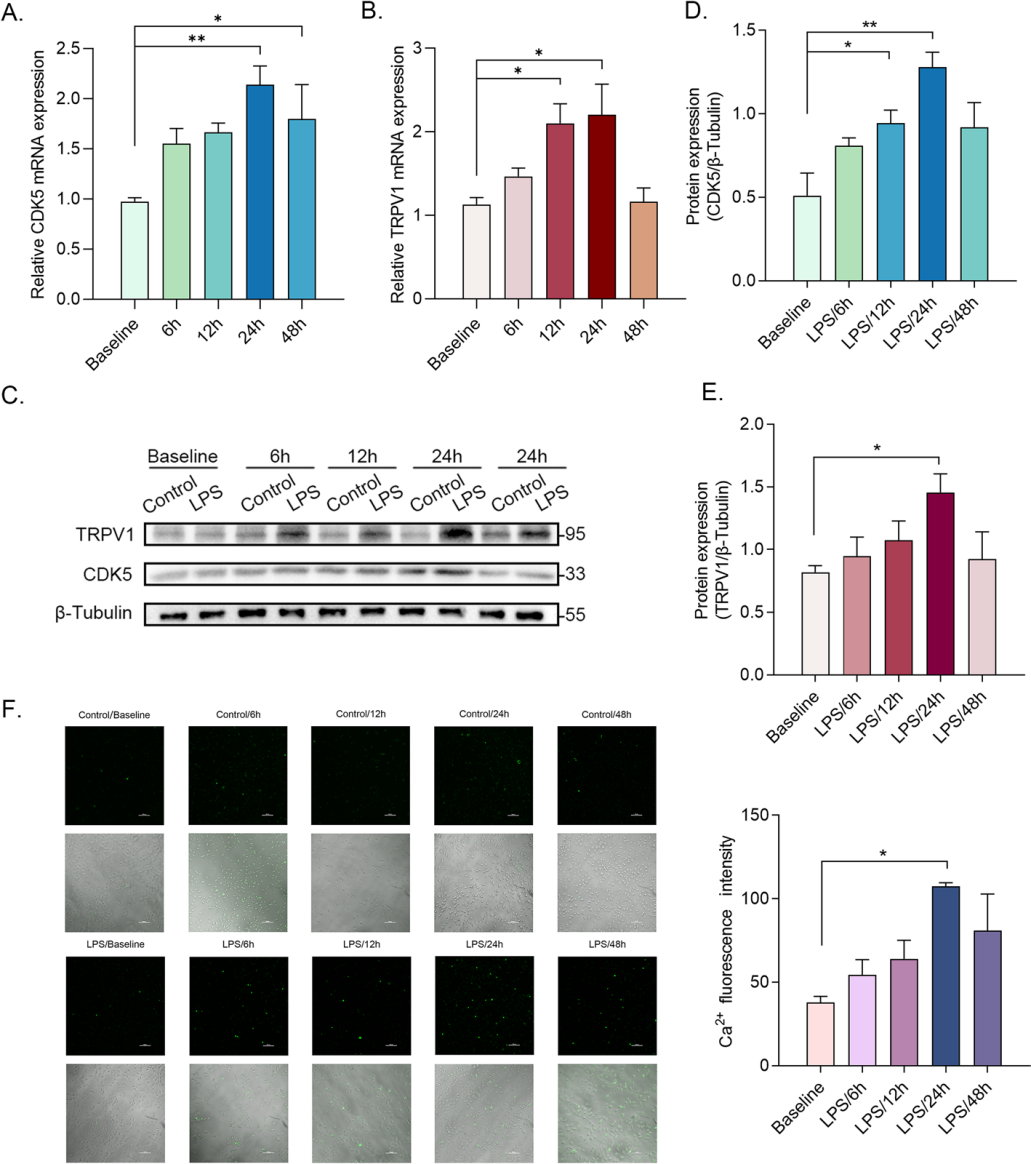
LPS upregulates the expression of TRPV1 and CDK5 in DRG neurons. **A and B** The relative expression mRNA levels of CDK5 (**A**) and TRPV1 (**B**) in DRG neurons after 6–48 h incubation with LPS (*n* = 3 per group). **C** Western blot results showed the protein expression of TRPV1 and CDK5 (*n *= 3 per group). **D** and **E** The relative protein expression levels of CDK5 (**D**) and TRPV1 (**E**) in DRG neurons after 6–48 h incubation with LPS. **F** Ca^2+^ signals in DRG neurons after a 6-48 h incubation with LPS (*n *= 3 per group) (scale bar, 100 μm). *p < 0.05; **p < 0.01; ***p < 0.0005; ****p < 0.0001

Based on the aforementioned in vivo findings, parallel in vitro experiments were conducted using DRG neurons to assess the presence of similar alterations. Rat DRG neurons were subjected to a concentration of 2 μg/ml of LPS for 6, 12, and 24 h, aiming to induce neuronal sensitivity changes, while monitoring the quantitative variations in CDK5 and TRPV1 protein and RNA expression levels at each distinct time interval (Fig. 2A–E) [26, 27]. An increasing trend was observed in both protein and RNA expression, which reached its maximal level 24 h after incubation with LPS. Previous investigations have validated the application of calcium imaging techniques for the purpose of detecting and assessing cellular excitability [28]. Therefore, we employed calcium imaging as a means to investigate the impact of varying LPS incubation duration on neuronal excitability. Remarkably, our findings revealed that intracellular calcium influx continued to peak even after 24 h of LPS incubation (Fig. 2F). The aforementioned cellular experiments provided evidence showing a positive relationship between the upregulation of CDK5 and TRPV1 expression and the elevated influx of Ca^2+^. Consequently, this implies a strong correlation between the expression of CDK5 and TRPV1 and the excitability of neurons.

### CDK5 mediates cell membrane transport of TRPV1 by phosphorylating at T406

**Fig. 3:**
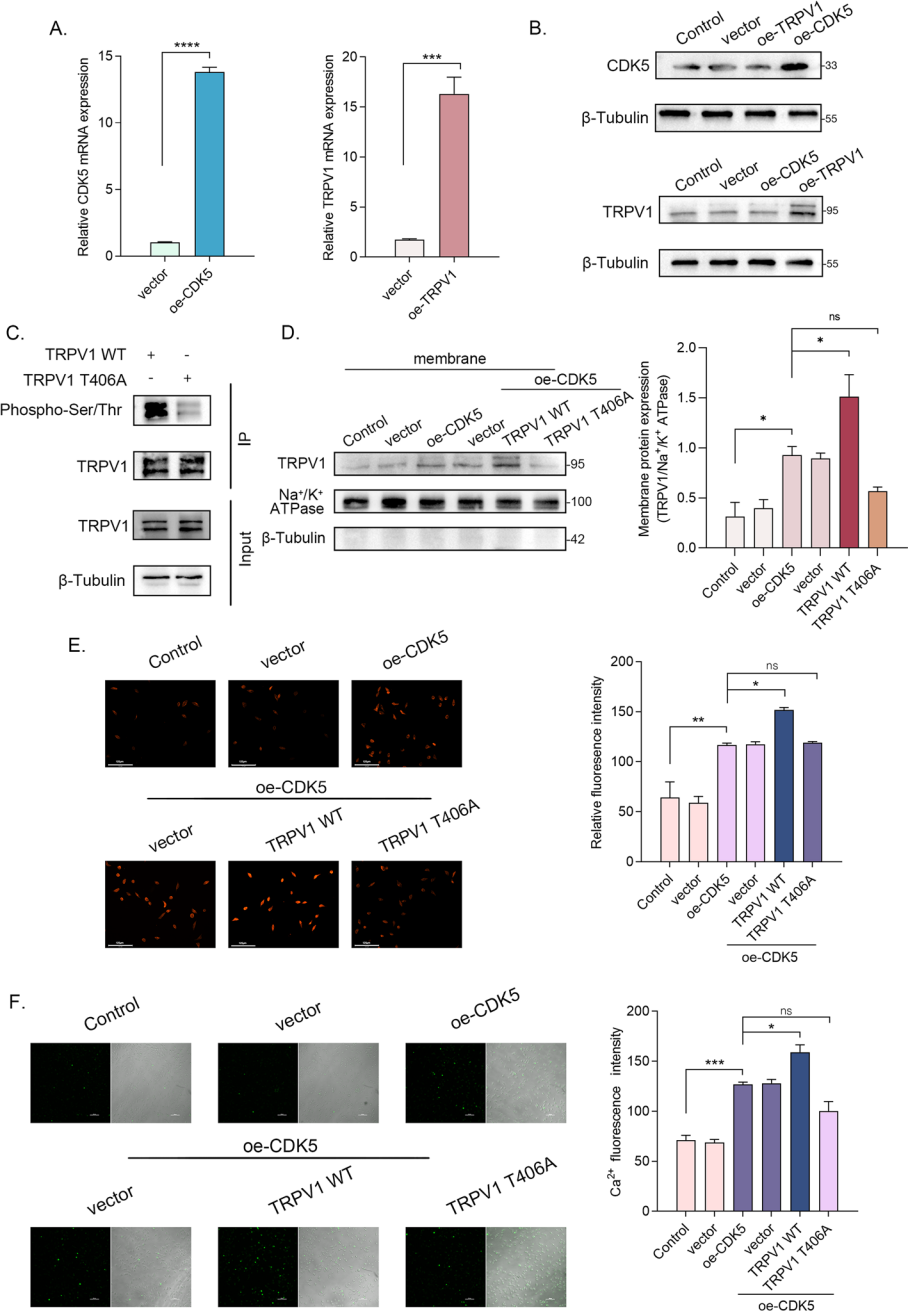
CDK5 mediates cell membrane transport of TRPV1 by phosphorylating at T406. **A** and **B** The relative mRNA and protein expression levels of TRPV1 and CDK5 in DRG neurons after transfection (*n* = 3 per group). **C** The phosphorylation levels of TRPV1 WT and TRPV1 T406A. **D** The relative expression levels of TRPV1 protein on DRG cell membrane after cotransfection with CDK5 and TRPV1 (WT/ T406A) (*n *= 3 per group). **E** TRPV1 (red fluorescence) stained in adherent DRG neurons with unruptured membranes after cotransfection with CDK5 and TRPV1 (WT/ T406A) (*n* = 3 per group) (scale bar, 125 μm). **F** Ca^2+^ signals in DRG neurons after cotransfection with CDK5 and TRPV1 (WT/ T406A) (*n *= 3 per group) (scale bar, 100 μm). ns, not statistically significant; *p < 0.05; **p < 0.01; ***p < 0.0005; WT, wild type

In a previous investigation, the process of membrane surface localization was observed to be facilitated by CDK5 through phosphorylation of TRPV1 [15]. The present study seeks to further elucidate the phosphorylation mechanism involving CDK5 and TRPV1 by conducting refined experiments using rat DRG neurons. To this end, we designed and constructed CDK5 and TRPV1 wild-type plasmids. The results of RT–qPCR and western blot confirmed the transfection efficacy and demonstrated an absence of mutual regulatory effects on protein expression levels between the two (Fig. 3A and B). To ascertain the phosphorylation sites of TRPV1, we generated a plasmid with a site mutation (T406A) in the TRPV1 coding region. Subsequently, we measured the broad phosphorylation levels in two groups of cells transfected with the wild-type and mutant TRPV1, respectively. The findings revealed a decrease in TRPV1 phosphorylation following mutation of the phosphorylation site (Fig. 3C). These experimental outcomes provide confirmation that the T406 site indeed serves as one of the phosphorylation sites of TRPV1, and the mutation at this site can impede the overall phosphorylation level of TRPV1.

To further confirm the role of CDK5 in promoting membrane transport through phosphorylation of TRPV1 at T406, we conducted experiments involving the separation of cell membranes and subsequent evaluation of TRPV1’s membrane localization level. The findings revealed that overexpression of CDK5 led to an increased membrane transport of TRPV1. Furthermore, overexpression of both CDK5 and wild-type TRPV1 resulted in a higher level of TRPV1 on the cell membrane when compared with simultaneous overexpression of CDK5 and TRPV1 T406A (Fig. 3D). To visually assess the extent of membrane transport, we performed immunofluorescence staining on both the cell membrane and total TRPV1, followed by merging to specifically label the TRPV1 protein localized on the membrane. To prevent binding to cytoplasmic TRPV1, we omitted the breaking of cell membrane during the immunofluorescence staining process. The results indicated that overexpression of CDK5 augmented the translocation of TRPV1 to the cell membrane. Co-overexpression with wild-type TRPV1 further amplified membrane-bound TRPV1, whereas co-overexpression with TRPV1 T406A exerted no such effect (Fig. 3E). Subsequently, calcium imaging was performed to assess cell excitability. Figure 3F depicts the overexpression of CDK5 enhanced Ca^2+^ influx; co-overexpression with wild-type TRPV1 further increased Ca^2+^ influx, while co-overexpression with TRPV1 (T406A) did not elicit a similar effect. The results demonstrated that CDK5 promoted TRPV1 membrane transport and neuron excitability through phosphorylation of TRPV1 at T406.

### Binding of TRPV1 and CDK5

**Fig. 4:**
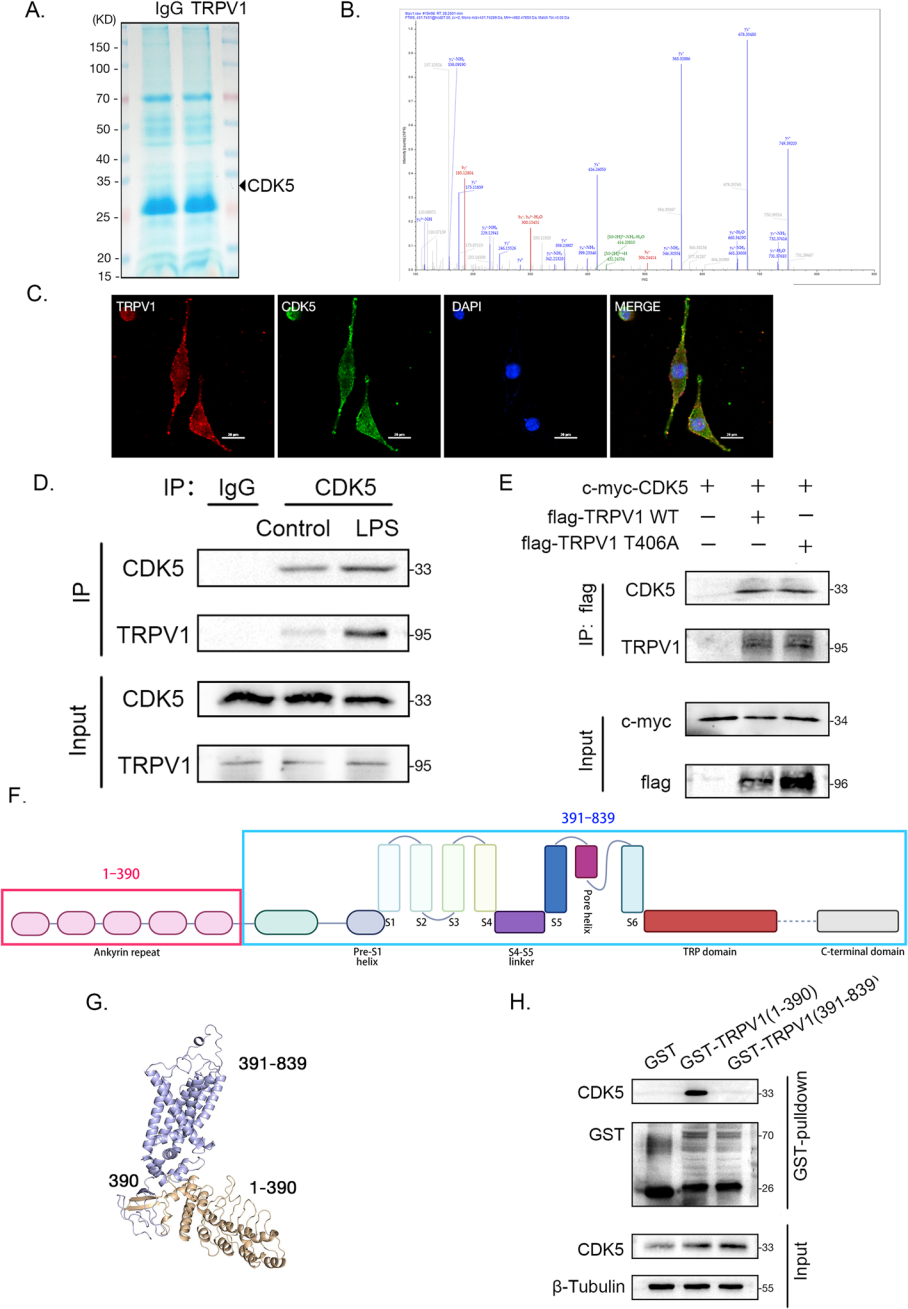
Binding of TRPV1 and CDK5. **A** Coomassie brilliant blue stained gels. **B** Mass spectrometry analysis of CDK5 binding to TRPV1. **C** CDK5 (green fluorescence), TRPV1 (red fluorescence), and DAPI (blue fluorescence) staining in DRG neurons (scale bar, 20 μm). **D** Co-IP was performed to identify the endogenous interaction between TRPV1 and CDK5 in DRG neurons. **E** Co-IP was performed to identify the exogenous interaction between TRPV1 and CDK5 in 293T cells. **F** Schematic representation of the linear structure and truncation of TRPV1. **G** Schematic representation of the spatial configuration and truncation of TRPV1. **H** GST-pulldown assay verified which truncated form of TRPV1 CDK5 bound to

While the phosphorylation of TRPV1 by CDK5 has been established, the precise binding mechanism between CDK5 and TRPV1 remains unclear. The proteins interacting with TRPV1 were detected by immunoprecipitation and mass spectrometry (Supplementary Table 1). Preliminary mass spectrometry results suggested that CDK5 might directly bind to TRPV1 (Fig. 4A and B). Bioinformatics analyses of the mass spectrometry results showed intracellular protein transport in biological processes (Supplementary Fig. 1). Moreover, immunofluorescence demonstrated potential colocalization of the two proteins (Fig. 4C). Subsequently, coimmunoprecipitation (co-IP) was conducted in DRG and 293T cell lines, respectively. The results in Fig. 4D and E demonstrate a binding interaction between CDK5 and TRPV1 in both cell lines, which was not contingent on the phosphorylation site T406. Thus, the binding region drew our attention. Referring to the major domains in the TRPV1 subunit observed in a previous study [29], we designed two truncated forms (1–390 and 391–839). The planar structures of the truncated forms are illustrated in Fig. 4F, while Fig. 4G represents their three-dimensional conformations. Prokaryotic plasmids encoding the truncated forms were constructed and transformed into *Escherichia coli *BL21 (DE3), and the truncated TRPV1 proteins were purified. Glutathione-*S*-transferase (GST) pulldown revealed a higher binding affinity of CDK5 toward TRPV1 (1–390) (Fig. 4H).

### MiR-142-5p targets both CDK5 and TRPV1

**Fig. 5:**
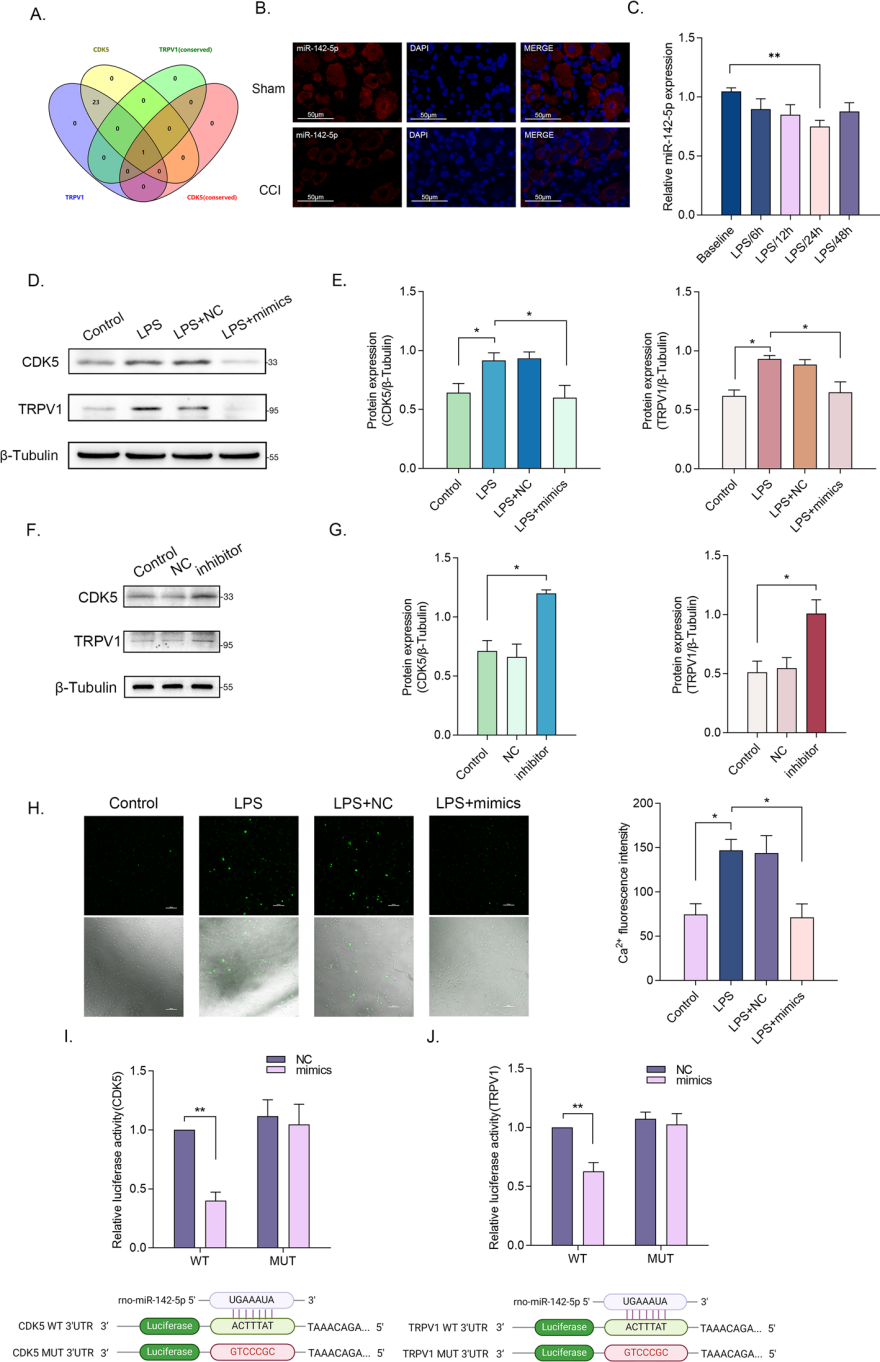
MiR-142-5p targets both CDK5 and TRPV1 **A** The predicted conserved miRNAs of CDK5 and TRPV1 in Targetscan database. **B** The expression of miR-142-5p in rat DRG tissues was determined by FISH assay. (scale bar, 125 μm). **C** The relative expression levels of miR-142-5p in DRG neurons after incubation with LPS for 6–48 h (*n *= 3 per group). **D** and **E** The relative protein expression levels of TRPV1 and CDK5 in DRG neurons after incubation with LPS and transfection with miR-142-5p mimics (*n *= 3 per group). **F** and **G** The relative protein expression levels of TRPV1 and CDK5 in DRG neurons after transfection with miR-142-5p inhibitor (*n *= 3 per group). **H** Ca^2+^ signals in DRG neurons after incubation with LPS and transfection with miR-142-5p mimics (*n *= 3 per group) (scale bar, 100 μm). **I** and **J** Dual-luciferase reporter gene assay. Luciferase activity was measured after co-transfection with the miR-142-5p mimic and the WT or MUT CDK5 (**I**) and TRPV1 (**J**) in 293T cells (*n *= 3 per group). *p < 0.05; **p < 0.01; ***p < 0.0005; ****p < 0.0001; WT, wild type

Numerous studies have confirmed the involvement of miRNAs in the regulation of NP. Thus, we explored the upstream miRNAs of CDK5 and TRPV1. By utilizing a bioinformatics website for upstream prediction, we made an intriguing discovery—a highly conserved miRNA, miR-142-5p, in both rats and humans exhibited significant conservation (Fig. 5A and Supplementary Tables 2 and 3). Based on this observation, we hypothesized that miR-142-5p might directly regulate the expression of TRPV1, and it might indirectly modulate the phosphorylation level and membrane transport state of TRPV1 by regulating CDK5 expression. FISH assay confirmed the presence of miR-142-5p in rat DRG tissues, and the expression of miR-142-5p decreased after the establishment of CCI model (Fig. 5B). The expression levels of miR-142-5p were detected by RT–qPCR at different LPS treatment times. It was evident that miR-142-5p exhibited a decreasing trend after LPS treatment, reaching its peak and nadir within 24 h (Fig. 5C). Next, miR-142-5p was overexpressed and knocked down to further investigate its regulatory function (Supplementary Fig. 2). Notably, the overexpression of miR-142-5p restored the elevated expressions of CDK5 and TRPV1 induced by LPS treatment (Fig. 5D and E), while miR-142-5p inhibitor promoted the expression of CDK5 and TRPV1 (Fig. 5F and G). Moreover, calcium imaging experiments conducted in the aforementioned groups corroborated our expectations, as miR-142-5p overexpression reversed the excitatory effects triggered by LPS treatment (Fig. 5H). Lastly, we conducted a dual-luciferase reporter gene assay to probe the regulatory effects of miR-142-5p on TRPV1 and CDK5 (Fig. 5I and J). Our findings indicated that miR-142-5p could bind to the wild-type TRPV1 and CDK5 but not to the mutant, thereby inhibiting luciferase activity. In summary, LPS treatment resulted in the downregulation of miR-142-5p, and miR-142-5p targeted both CDK5 and TRPV1 by binding to their 3′UTRs.

### MiR-142-5p works along both lines to reduce membrane localization of TRPV1

**Fig. 6:**
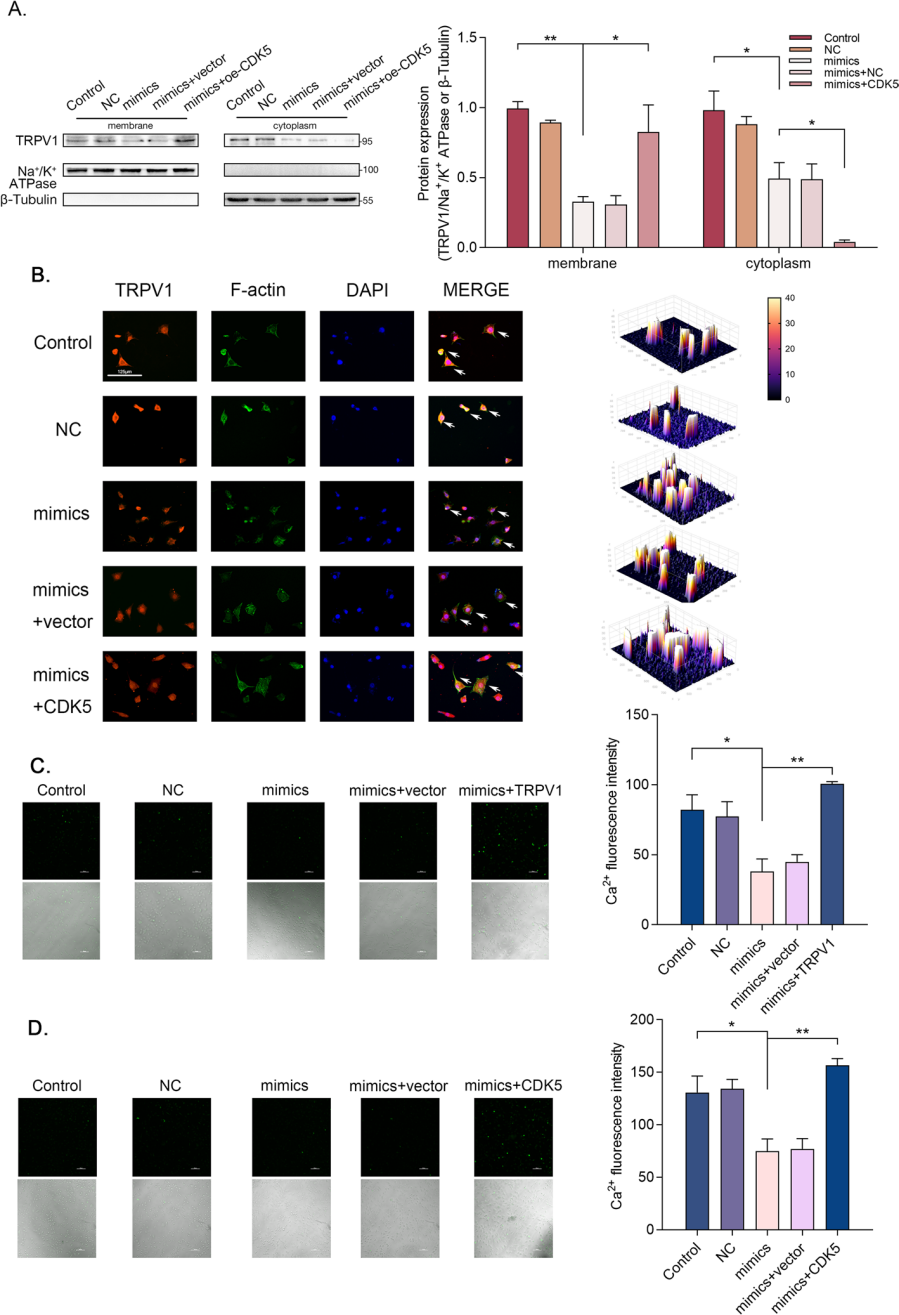
MiR-142-5p works along both lines to reduce membrane localization of TRPV1 **A** The relative protein levels of TRPV1 located in DRG cell membrane and cytoplasm after cotransfection with miR-142-5p mimics and CDK5 (*n *= 3 per group). **B** Staining of TRPV1 (red fluorescence), filamentous actin (green fluorescence), and DAPI (blue fluorescence) in paraffin sections of DRG neurons after co-transfection with miR-142-5p mimics and CDK5 (scale bar, 125 μm). **C** and **D** Ca^2+^ signals in DRG neurons after cotransfection with miR-142-5p mimics and TRPV1 (**C**) or CDK5 (**D**) (*n *= 3 per group) (scale bar, 100 μm); ns, not statistically significant; *p < 0.05; **p < 0.01

To evaluate the effects of miR-142-5p on the membrane localization of TRPV1, cell membrane and cytoplasmic separation technique were employed. The results revealed that miR-142-5p negatively regulated the overall abundance and membrane expression of TRPV1, whereas overexpression of CDK5 rescued TRPV1 membrane expression and concurrently decreased its cytoplasmic expression (Fig. 6A). The immunofluorescence findings were consistent with the aforementioned western blot results (Fig. 6B). Collectively, miR-142-5p did not merely exert a direct negative effect on TRPV1 expression but indirectly suppressed the membrane trafficking of TRPV1 by inhibiting CDK5 expression. Subsequently, calcium imaging was conducted to explore the underlying mechanism of miR-142-5p on the cell excitability. The results demonstrated that miR-142-5p induced a significant reduction in Ca^2+^ inflow; however, co-overexpression of CDK5 or TRPV1 substantially reversed the decrease in calcium inflow (Fig. 6C and D).

### Methyltransferase-like 14 (METTL14) decelerates the maturation of pri-miR-142 in an m^6^A dependent manner

**Fig. 7:**
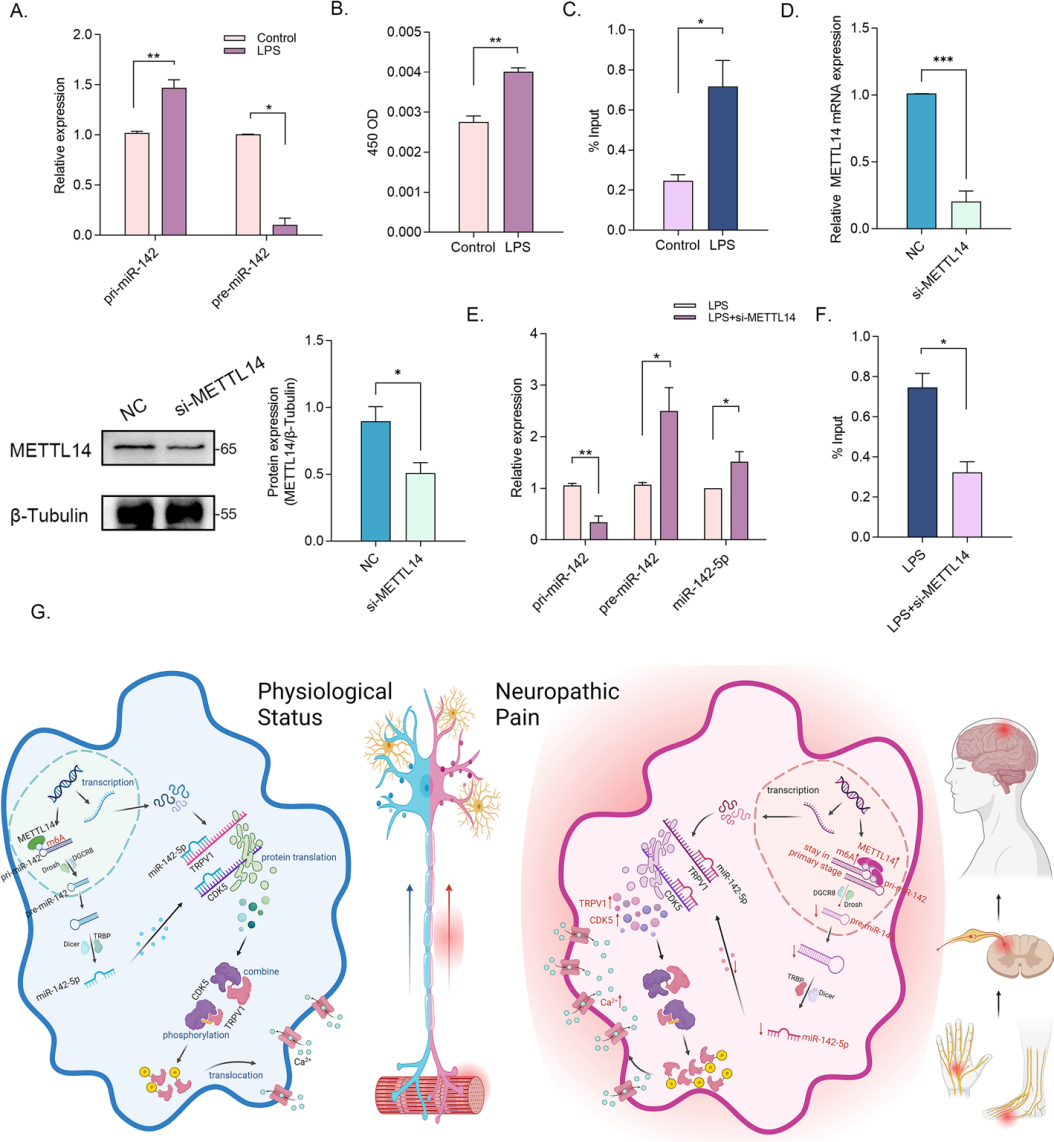
METTL14 decelerated the maturation of pri-miR-142 in an m^6^A dependent manner **A** The relative expression levels of pri-miR-142 and pre-miR-142 in DRG neurons after 24 h incubation with LPS (*n *= 3 per group). **B** m^6^A RNA methylation colorimetric quantification assay measured the total m^6^A modification level after 24 h incubation with LPS (*n *= 3 per group). **C** MeRIP assay determined the methylation levels of pri-miR-142 after 24 h incubation with LPS (*n *= 3 per group). **D** The relative protein and mRNA expression levels of METTL14 in DRG neurons after transfection with si-METTL14 (*n *= 3 per group). **E** The relative expression levels of pri-miR-142, pre-miR-142, and miR-142-5p in DRG neurons after 24 h incubation with LPS and transfection with si-METTL14 (*n *= 3 per group). **F** MeRIP assay determined the methylation levels of pri-miR-142 after 24 h incubation with LPS and transfection with si-METTL14 (*n *= 3 per group). *p < 0.05; **p < 0.01 **G** The schematic diagram illustrating the proposed mechanism. The figure was created with Biorender.com

To further elucidate the causes of reduced expression of miR-142-5p in LPS-treated DRG, the expression changes of pri-miR-142, pre-miR-142, and miR-142-5p were examined. Surprisingly, we observed an increase in pri-miR-142 expression, while the expressions of pre-miR-142 and miR-142-5p were decreased (Figs. 5C and7A). This suggested that the maturation of pri-miR-142 to pre-miR-142 was inhibited. Previous studies have indicated that m^6^A modification of RNA can influence miRNA maturation process [30]. Therefore, we investigated whether the inhibited miRNA maturation was related to the alterations in m^6^A levels. As expected, we found an upregulation of overall m^6^A levels induced by LPS (Fig. 7B). Furthermore, methylated RNA immunoprecipitation (meRIP) assay revealed a significant increase in m^6^A modification of pri-miR-142 after LPS treatment (Fig. 7C). Subsequently, the expressions of three common m^6^A methyltransferases were analyzed, and the most prominent difference was shown in the expression level of METTL14 induced by LPS (Supplementary Fig. 3). The knockdown efficiency of METTL14 was verified (Fig. 7D), and meRIP results demonstrated a significant downregulation of pri-miR-142 methylation levels after METTL14 knockdown (Fig. 7E). Meanwhile, METTL14 knockdown led to increased expression levels of pre-miR-142 and mature miR-142-5p, while pri-miR-142 expression decreased (Fig. 7F). Collectively, it was evident that METTL14 could decelerate the maturation process of pri-miR-142 in an m^6^A-dependent manner, leading to a reduction in miR-142-5p expression. Figure 7G illustrated this comprehensive pathway, spanning from nuclear signaling to membrane function.

### MiR-142-5p plays a protective role in NP in vivo

**Fig. 8:**
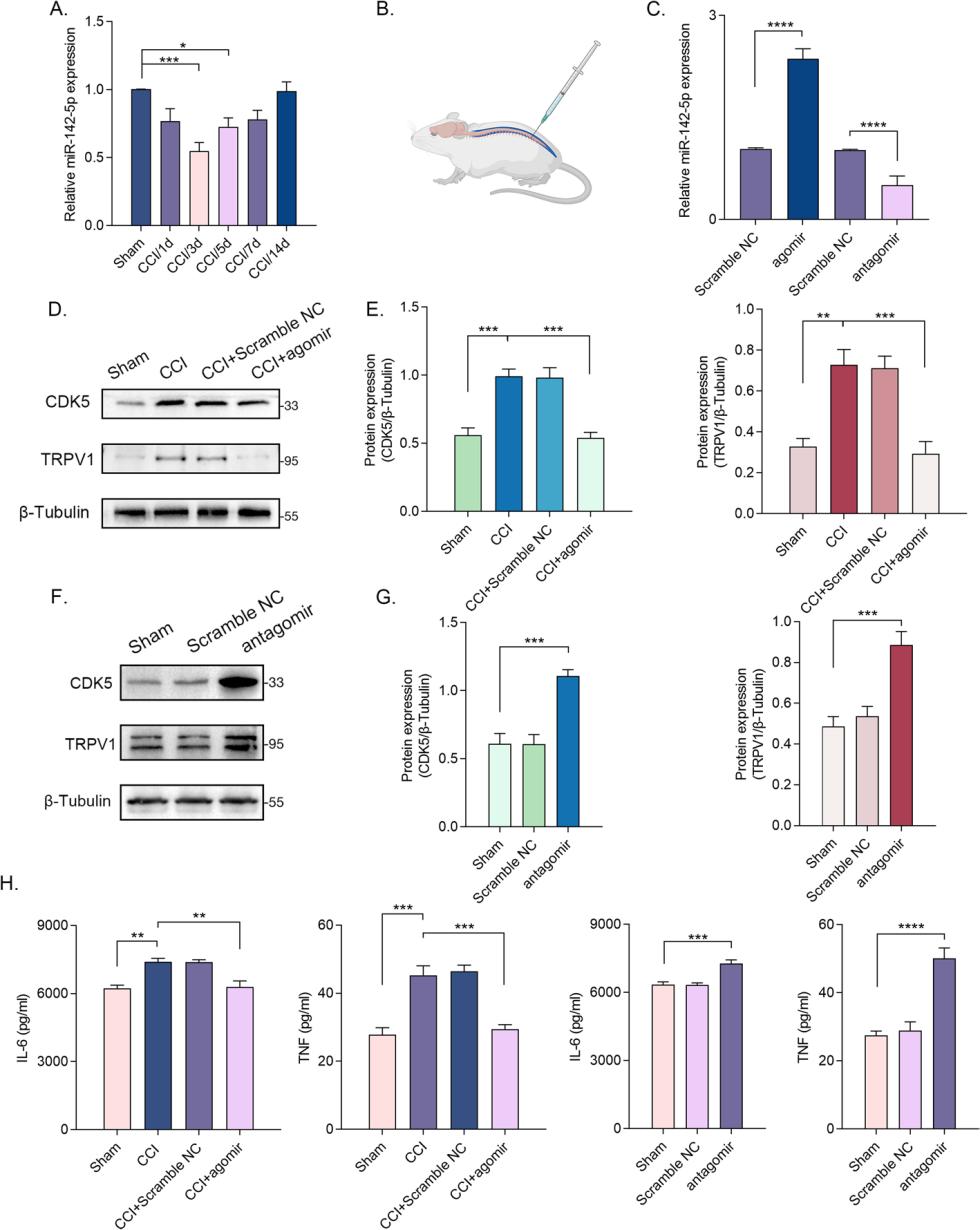
MiR-142-5p plays a protective role in NP in vivo **A** The relative expression levels of miR-142-5p in DRG tissue at 1–14 days after CCI (*n *= 5 per group). **B** Schematic of the intrathecal injection in the rat. The figure was created with Biorender.com. **C** The relative expression levels of miR-142-5p in DRG tissue after the intrathecal injection of miR-142-5p agomir or antagomir (*n *= 5 per group). **D** and **E** The relative protein expression levels of CDK5 and TRPV1 in DRG tissue after CCI and intrathecal injection of miR-142-5p agomir (*n *= 5 per group). **F** and **G** The relative protein expression levels of CDK5 and TRPV1 in DRG tissue after intrathecal injection of miR-142-5p antagomir (*n *= 5 per group). **H** The relative expression levels of IL-6 and TNF in DRG tissue after CCI and intrathecal injection of miR-142-5p agomir or antagomir (*n *= 5 per group). ns, not statistically significant; *p < 0.05; **p < 0.01; ***p < 0.0005; ****p < 0.000

The expression of miR-142-5p in DRG tissue was continuously monitored subsequent to the establishment of CCI model. The results obtained from RT–qPCR analysis revealed that the expression of miR-142-5p was found to be at its minimum level at 3 days post-injury (Fig. 8A). To ascertain the effects of miR-142-5p on NP in vivo, we modulated the expression of miR-142-5p by administering agomir or antagomir via intrathecal injection for three consecutive days (Fig. 8B). The efficiency of intrathecal injection and transfection was validated (Fig. 8C). The expression levels of CDK5 and TRPV1 in DRG were analyzed following the intrathecal injection of agomir or antagomir. The results demonstrated that miR-142-5p agomir inhibited the CCI-induced upregulation of CDK5 and TRPV1; on the contrary, miR-142-5p antagomir promoted the expression of CDK5 and TRPV1 in the sham rats (Fig. 8D–G). In addition, we assessed the expression of inflammatory factors and behavioral responses. Intrathecal administration of miR-142-5p agomir exhibited therapeutic effects, while intrathecal administration of miR-142-5p antagomir induced inflammatory responses and hyperalgesia (Fig. 8H and Supplementary Fig. 4). Therefore, miR-142-5p was found to influence the progression of NP, and it might be a potential therapeutic target.

## Discussion

NP can arise following nerve injury, characterized by detrimental changes in injured neurons, nociceptive pathways and descending modulatory pathways within the central nervous system. The myriad neurotransmitters and other substances involved in the development and maintenance of NP also play a part in other neurobiological disorders. Pharmacological and genetic studies confirm that TRPV1 plays a role in a variety of pathological pain models. TRPV1 channels are sensitive ion channels activated by a number of noxious stimuli that alter many physiological functions upon activation. Pharmacological and genetic studies confirm that TRPV1 plays a role in a variety of pathological pain models [31–33]. Extensive investigation of TRPV1 in NP has become predominant [32, 34]. TRPV1 activity is primarily associated with the detection of noxious heat and the induction of inflammatory hyperalgesia [35]. Emerging studies suggest that protein kinases modulate the phosphorylation levels of pain-signaling proteins, potentially contributing to NP development [36]. Among multiple kinases activated in response to inflammation or nerve injury, CDK5 is noteworthy. Under conditions of neurotoxic stress, inflammation, or neuronal injury, the proteolytic cleavage of CDK5 activators p35 and p39 by calpain, in response to increased calcium influx, generates p25 and p29, respectively. The subsequent formation of CDK5/p25 or CDK5/p29 complexes allows for their cytoplasmic translocation, leading to the phosphorylation of diverse cellular targets [14]. In this study, we investigate the direct binding mechanism and the binding site between TRPV1 and CDK5 for the first time. Furthermore, we discovered that miR-142-5p not only directly regulated the expression of TRPV1 but also indirectly modulated the phosphorylation and membrane transport of TRPV1 by regulating CDK5 expression. In addition, we proposed an innovative role for METTL14 in mediating the biosynthesis of miR-142-5p in an m^6^A dependent manner.

Previous studies have shown that CDK5 reduces the interaction of TRPV1 with AP2μ2 by phosphorylating AP2μ2, which in turn mediates reduced internalization of TRPV1 [37]. CDK5 also directly phosphorylates TRPV1 and upregulates the cell membrane surface localization of TRPV1 [15]. In previous studies of CDK5 phosphorylation of TRPV1, the F11 cell line was used [15, 38]. The F11 cell line, derived from the fusion of a rat embryonic DRG and a mouse neuroblastoma cell line (NT18TG2), retains the chromosomes of both rat and mouse. To avoid this problem, the cells used in the in vitro experiments were entirely sourced from rats. Furthermore, calcium imaging experiments were implemented to observe the alterations of cellular excitability more directly. We devised the CDK5 plasmid and carried out its transfection, which led to a significant augmentation in the localization of TRPV1 on the cell membrane and a significant reduction in its localization in the cytoplasm. Subsequently, the interaction between CDK5 and TRPV1 was investigated, revealing that CDK5 regulates membrane transport through the phosphorylation of TRPV1 at T406. Upon transfection of the TRPV1 (T406A) plasmid into DRG cells, we isolated the plasma membrane and detected a marked decrease in the membrane localization of TRPV1. Importantly, an in-depth investigation was conducted on the binding domain of CDK5 and TRPV1. By analyzing the TRPV1 protein structure domains and the spatial conformation, two truncated forms were designed, highlighting a preference for CDK5 binding with the 1–390 region of the truncated protein. Interestingly, the binding of CDK5 to TRPV1 is independent of the phosphorylation site T406. In the future, new drugs that target the phosphorylation site and the binding sites are expected to become new stars in the treatment of NP.

In numerous research studies, the exploration of miRNA regulation on individual genes is typically carried out. Nevertheless, it is crucial to acknowledge that regulatory relationships within organisms frequently form intricate networks or display diverse patterns. In this breakthrough finding, miR-142-5p was identified as the sole highly conserved miRNA capable of concurrently regulating two downstream targets, namely CDK5 and TRPV1. The inhibitory effects of miR-142-5p on mRNAs were affirmed by luciferase reporter assays. Additionally, the reversal experiments provided evidence that miR-142-5p suppressed the excitability of DRG neurons by acting on TRPV1 and CDK5. Subsequently, we innovatively found that the expression of TRPV1 was directly targeted and regulated by miR-142-5p and also that the phosphorylation and membrane transport of TRPV1 were indirectly regulated by miR-142-5p via CDK5.

Previous studies have primarily focused on the transcriptional regulation of miRNA expression. However, our findings in this study have revealed an unexpected inconsistency between the expression of pri-miR-142 and its corresponding pre-miR-142 and mature miR-142-5p. This observation strongly suggested that the differences in miR-142-5p expression induced by LPS are primarily attributed to the maturation process of pri-miR-142, rather than transcriptional regulation. The initial step in miRNA biosynthesis involves the processing of primary miRNAs (pri-miRNAs), which are cut into hairpin-structured precursor miRNAs (pre-miRNAs) by the nuclear microprocessor complex containing Drosha and DiGeorge critical region 8 (DGCR8) [39]. It is worth noting that the maturation of miRNAs is a complex process regulated by various factors including DROSHA cleavage, exportin-5 trafficking, and DICER cleavage. In mammalian mRNA, m^6^A modification is a prevalent post-transcriptional modification. Recent advancements in high-throughput sequencing techniques have revealed widespread *N*^6^-methylation of pri-miRNAs, in addition to mRNA. In this study, we observed an increase in the overall m^6^A level and m^6^A modification, specifically within pri-miR-142 following LPS treatment. Methyltransferases, such as METTL14 and methyltransferase-like 3 (METTL3), have been identified to methylate pri-miRNAs, thereby facilitating their recognition and processing by DGCR8 [40, 41]. In the present study, METTL14 exhibited the most significant upregulation in response to LPS treatment. Knockdown of METTL14 resulted in decreased methylation levels and promoted the maturation of pri-miR-142.

In conclusion, we present novel findings regarding the binding domain of TRPV1 with CDK5 and the independent nature of their interaction from the phosphorylation site. The results in the present study suggest potential therapeutic implications in the clinical translation of TRPV1 modulation. Specifically, we proposed the direct inhibition of CDK5-mediated phosphorylation as a means to attenuate NP, as well as the indirect modulation of TRPV1 phosphorylation by targeting their binding regions. Moreover, the dual mechanisms of miR-142-5p for NP remission were demonstrated. Not only did it directly regulate TRPV1 expression but also modulated TRPV1 membrane transport through regulating CDK5 expression. In addition, we elucidated the inhibitory effects of METTL14 on the biosynthesis of miR-142-5p in an m^6^A-dependent manner. Given the favorable potency of miR-142-5p, enhancing endogenous miR-142-5p expression or supplementing exogenous miR-142-5p via exosomes and other pathways will be new approaches to treat NP.

## Conclusions

Within this investigation, our research delineated a pronounced increase in TRPV1 channels and CDK5 expression within the DRG tissue of CCI model rats and DRG neurons subjected to LPS. Noteworthy findings demonstrate that the phosphorylation of TRPV1 was modulated by CDK5, thus orchestrating the membrane trafficking of TRPV1. Through methodologies such as immunofluorescence and membrane-cytoplasm fractionation in western blot analyses, the translocation of TRPV1 to the membrane was clearly illustrated. The interplay between TRPV1 and CDK5, alongside their binding sites, was also elucidated. Our analysis and research reveal that miR-142-5p operated as a pivotal upstream regulator, exerting inhibitory influences on the expression levels of both CDK5 and TRPV1. Furthermore, our observations suggest that METTL14 facilitates m^6^A modification of primary miR-142 in the CCI model, impeding the maturation process of primary miR-142 and consequently causing a reduction in mature miR-142-5p levels. The interaction between CDK5 and TRPV1, recognized as a key factor facilitating TRPV1 membrane translocation, indicates that synthetic short peptides rivaling CDK5 for the binding to TRPV1 might represent highly promising therapeutic modalities for managing NP.

## Supplementary Information

Additional file 1. Table 1. List of TRPV1-interacting proteins identified by co-immunoprecipitation and mass spectrometry. Table 2. List of upstream miRNAs of TRPV1 were predicted by Targetscan. Table 3. List of upstream miRNAs of CDK5 were predicted by Targetscan. Table 4. Primers for RT–qPCR analysis. Table 5. List of Antibodies. Table 6. List of Plasmid. Figure 1. GO and KEGG. Figure 2. Efficiency of mimics and inhibitor. Figure 3. Expressions of three common m^6^A methyltransferases. Figure 4. Paw withdrawal mechanical threshold and paw withdrawal thermal latency in rats after intrathecal injection of agomir.

## Data Availability

The datasets generated during and/or analyzed during the current study are available from the corresponding author on reasonable request.

## Abbreviations

NP: Neuropathic pain
CDK5: Cyclin-dependent kinase 5
TRPV1: Transient receptor potential vanilloid 1
m^6^A: *N*^6^-methyladenosine
PWMT: The paw withdrawal mechanical threshold
PWTL: Paw withdrawal thermal latency
METTL14: Methyltransferase-like 14
DRG: Dorsal root ganglion
TRP: Transient receptor potential
miRNAs: MicroRNAs
CCI: Chronic constriction injury
FBS: Fetal bovine serum
HBSS: Hank’s balanced salt solution
PMSF: Phenylmethylsulfonyl fluorid
RIPA: Radioimmunoprecipitation assay
PVDF: Polyvinylidene fluoride
RT–qPCR: Real-time quantitative reverse transcription PCR
LB: Luria–Bertani
IPTG: Isopropyl β-d-1-thiogalactopyranoside
co-IP: Coimmunoprecipitation
MeRIP: Methylated RNA immunoprecipitation
ELISA: Enzyme-linked immunosorbent assay
TMB: 3,3′,5,5′-Tetramethylbenzidine
LPS: Lipopolysaccharide
METTL3: Methyltransferase-like 3
pri-miRNAs: Processing of primary miRNAs
pre-miRNAs: Precursor miRNAs
DGCR8: DiGeorge critical region 8
