# Supplementary material for: Biosynthesis inhibition of miR-142-5p in a N6-methyladenosine-dependent manner induces neuropathic pain through CDK5/TRPV1 signaling
Source: Cell Mol Biol Lett. 2025 Jan 31;30:16. doi: 10.1186/s11658-025-00695-w (PMC11786349; doi:10.1186/s11658-025-00695-w)
Supplement: Supplementary file 1 — Additional file 1. Table 1. List of TRPV1-interacting proteins identified by co-immunoprecipitation and mass spectrometry. Table 2. List of upstream miRNAs of TRPV1 were predicted by Targetscan. Table 3. List of upstream miRNAs of CDK5 were predicted by Targetscan. Table 4. Primers for RT–qPCR analysis. Table 5. List of Antibodies. Table 6. List of Plasmid. Figure 1. GO and KEGG. Figure 2. Efficiency of mimics and inhibitor. Figure 3. Expressions of three common m6A methyltransferases. Figure 4. Paw withdrawal mechanical threshold and paw withdrawal thermal latency in rats after intrathecal injection of agomir. [file 11658_2025_695_MOESM1_ESM.pdf]

# Supplementary Materials for

## Exploring the Role of m6A Modification in the miR-142-5p/CDK5/TRPV1 Axis: Implications for Neuropathic Pain

**Jinshi Li et al.**

**Corresponding author: Bo Fang**

**This PDF file includes:**

**Supplementary Table 1. List of TRPV1-interacting proteins identified by co-immunoprecipitation and mass spectrometry (Page 1-33)**
**Supplementary Table 2. List of upstream miRNAs of TRPV1 predicted by Targetscan (Page 34-38)**
**Supplementary Table 3. List of upstream miRNAs of CDK5 predicted by Targetscan (Page 39-40)**
**Supplementary Table 4. Primers for RT-qPCR analysis (Page 41)**
**Supplementary Table 5. List of Antibodies (Page 42)**
**Supplementary Table 6. List of Plasmid (Page 43)**
**Supplementary Figure 1. GO and KEGG (Page 44-45)**
**Supplementary Figure 2. Efficiency of mimics and inhibitor (Page 46)**
**Supplementary Figure 3. Expressions of three common m6A methyltransferases (Page 47)**
**Supplementary Figure 4. Paw withdrawal mechanical threshold and paw withdrawal thermal latency in rats after intrathecal injection of agomir (Page 48)**

**Supplementary data**

**Supplementary Table 1. List of TRPV1-interacting proteins identified by co-immunoprecipitation and mass spectrometry**

| IgG | TRPV1 | | | |
|---|---|---|---|---|
| Dnm2 | Dnajb11 | Ctsb | Prss1 | Mtx2 |
| Actn1 | Dlat | Fbxo6 | Npm3 | LOC103694226 |
| Fah | Ndufa9 | Cav1 | Acadl | Apobec3 |
| Gns | Mthfd1 | Ppp6c | Hagh | Rab1a |
| Dars1 | Cbx3 | Tdrd7 | Asph | Agap1 |
| Dpysl2 | Dnm2 | Mrpl30 | Acat1 | Ppil1 |
| Psma2 | Actn1 | Tfb1m | Upf1 | Snrpn |
| C3 | Aldh5a1 | Pygb | Eif2b1 | Me2 |
| Mfge8 | Fh | Slc25a12 | Tmed9 | Lsm8 |
| Copb2 | Fah | Atp9b | Capza2 | Linc00176 |
| Coro6 | Gns | Wdr7 | Col8a1 | Plod3 |
| Epdr1 | Fgb | Rpl3 | Ap1g1 | Ldhb |
| Dnaja2 | Vps11 | Tpp1 | Gbf1 | Lamp2 |
| Mcpt9 | Dars1 | Pabpc4 | Rap1a | Cyp51a1 |
| Casp1 | Gpd2 | Pgm1 | Ap2s1 | Xpnpep1 |
| Hint1 | Cotl1 | Gtf3c1 | Rab3gap2 | Ncln |
| Adh7 | Eif2b4 | F11r | Psat1 | Lap3 |

| | | | | |
|---|---|---|---|---|
| Ahnak | Fbn1 | Dcaf13 | Soat1 | Nnt |
| Isyna1 | Dpysl2 | Phka2 | Tpp2 | Etfdh |
| Anxa2 | Gna13 | Acsf2 | Inppl1 | Mrpl19 |
| ENSRNOG00000067273 | Psma2 | Rragd | Gsn | Fryl |
| Akr1a1 | Dnajc13 | Park7 | Kifbp | Fcgbp |
| Adh4 | C3 | Gba | Rpl32 | Rack1 |
| Ctcf | Rps8 | Bsg | Pitrm1 | Ptpn9 |
| Ppm1g | Bphl | Cad | Tmem256 | Ckap5 |
| Rpl18a | Mfge8 | Tmem33 | Gaa | Zbtb8os |
| Hebp1 | Copb2 | Angptl2 | Cndp2 | Xpnpep3 |
| Cd47 | Rab10 | AABR07007121.1 | Gapvd1 | Mboat7 |
| Ddb1 | Rig1 | Rps6 | Pdia6 | Utp23 |
| Igh-1a | Adck1 | Ap3m1 | Acsbg1 | Rnpep |
| Atp5f1a | Ldlrap1 | Gtf3c2 | Osbpl3 | Ganab |
| RT1-A1n | Rbx1 | Ywhab | Eif3c | Dhx15 |
| Tmprss13 | Ankrd28 | Psmc1 | Ubr4 | Snx8 |
| Copa | Atp5pb | Sf3b1 | Rps25 | Pdpr |
| Ppia | Rars1 | ND1 | Kcnab2 | Chd4 |
| Pfdn1 | Abcb7 | Xpo5 | Alg5 | Sntb2 |
| LOC500180 | Hadhb | LOC500712 | Eif5a | ENSRNOG00 |

| | | | | |
|---|---|---|---|---|
| | | | | 000068034 |
| Shmt1 | Gyg1 | Eif4a1 | Ogdh | Smpd1 |
| Rps15al4 | Slc35b1 | Tmx1 | Hs2st1 | Tmed2 |
| Myof | Slfn13 | Polr1b | Pten | Ddost |
| Galm | Epdr1 | Msn | Uqcrc2 | Echs1 |
| Gstm2 | Dnaja2 | Mnda | Nup214 | Rpia |
| Mroh6 | Mcpt9 | Xrn2 | ENSRNOG00 000062879 | Lrrc59 |
| ENSRNOG00 000070978 | P4ha1 | Parp1 | Jak1 | Itgb5 |
| Arf3 | Naa50 | Stat3 | Prkaca | Cacybp |
| Selenbp1 | Parva | Braf | Rsl1d1 | Trpv1 |
| Galnt2 | Rps6ka6 | Smc5 | Ampd2 | Kat7 |
| Arsb | Casp1 | Tln1 | Lsm3 | Erlin2 |
| Aldoc | Adam17 | Prkcd | LOC367586 | Stt3b |
| Ripor2 | Vps13c | Psmb10 | AABR070369 74.1 | Dstn |
| Psmd13 | Edc4 | Mat2a | Krt14 | LOC10834969 1 |
| RGD1564606 | Adh7 | Actr2 | Ifitm2-ps2 | Arl2bp |
| Gdi1 | Usp9x | Ttc27 | Blmh | Cox15 |
| Rps13 | Rpl5 | Snrpa | Myo5a | Ppa1 |

| | | | | |
|---|---|---|---|---|
| Arf4 | Rap1b | Fkbp1a | Rdx | Nudt13 |
| ENSRNOG00000068118 | Dym | Dpt | Pnpt1 | Eif2s2 |
| Rpn2 | Kif2a | Ptpmt1 | Grn | Rad50 |
| Cisd1 | Ahnak | Ppif | Tm9sf4 | Zmpste24 |
| Naglu | Ctsa | Lclat1 | Tufm | Uap1l1 |
| Cdh26 | Isyna1 | Hspa4 | Tmem205 | Taok1 |
| Lonp1 | Nipsnap3a | Arl2 | Hic2 | Fitm2 |
| Acot1 | Cisd3 | Rpl10 | Bop1 | Htra2 |
| Atic | Pigt | Etfa | LOC681314 | Smchd1 |
| Rpl26 | Pdha1 | Prpf19 | Skic3 | Krt10 |
| Eif2a | Anxa2 | Tmem214 | Naa15 | Aaas |
| Ahcyl1 | Mogat2 | Lgals9 | LOC100912027 | Msh2 |
| Prdx1l1 | Lpcat3 | Tcf25 | Ube2h | Alg2 |
| Pkm | Ndufa8 | Smc3 | Armc8 | Snx9 |
| Alad | Pfdn5 | Fbl | Ankrd17 | Hacd2 |
| LOC500959 | Akr1a1 | Psmb4 | Acot9 | Syncrip |
| Hnrnpa1 | Cars1 | Ethe1 | Npepl1 | Cd63 |
| G3bp1 | Abcd3 | Tm9sf3 | Vps4b | Hsph1 |
| Pgls | Rab33b | Fggy | Zc3h7a | Apmap |
| Psmb2 | Rdh11 | Psmd7 | Hspa8 | Ptma |

| | | | | |
|---|---|---|---|---|
| Rbbp4 | Adh4 | Skp1 | Lamtor3 | Nup155 |
| LOC679748 | G3bp2 | Psma5 | Chd7 | Puf60 |
| Ywhaq | Psmc6 | Sparc | Mapk10 | Mitd1 |
| Tsn | Ces2 | Chrng | Taldo1 | Pura |
| Tll1 | Myo1d | Supt5h | Srp9 | Gar1 |
| Hspa5 | Ppm1g | Crat | Fcsk | Trip12 |
| Coq9 | Pdgfrb | Colgalt1 | Ltn1 | Dpp7 |
| Nptn | Tfrc | Twf1 | Hnrnpll | Steap2 |
| Nudcd1 | Capza1 | Rbbp5 | Frrs1 | Elob |
| ENSRNOG00000064589 | Rpl18a | Loxhd1 | Ssbp1 | Ephx1 |
| Lmnb1 | Hebp1 | Lancl2 | 0 | Folr1 |
| Fasn | Pcna | Vcl | Krt2 | Slc3a2 |
| Mesd | Psmb5 | Glmn | Samd9l | Glud1 |
| Cpt2 | Cmbl | Sf3a1 | Pepd | Txn2 |
| Rps16 | Tmem41b | ENSRNOG00000065655 | Wdr1 | Tubb6 |
| Timm13 | Cd47 | Scpep1 | Oscp1 | Opa1 |
| Aars1 | Plekho1 | Psmc3 | LOC679899 | Gpx4 |
| Tnpo1 | Hnrnpd | Eif3d | LOC100361060 | Mms19 |
| Eloc | Osbpl2 | Cct6a | Sec13 | Nfkb1 |

| | | | | |
|---|---|---|---|---|
| Cfl1 | Ddb1 | Igf2 | Ap2b1 | Rab35 |
| Napsa | Haus6 | Slc25a24 | Capzb | Atp1a1 |
| Eif3k | Gapdh | Celf1 | Septin8 | Papss1 |
| Lgals1 | Igh-1a | Celsr3 | Pcyox1l | Tor1b |
| Canx | Fermt2 | Ppp1ca | Prps1 | Fastkd5 |
| LOC103690796 | Atp5f1a | Asap1 | Sfpq | Abcb1a |
| Cpox | Drg2 | Insrr | Nup98 | Polr2h |
| Aldh1l2 | Tmem181 | Nup205 | Cpsf6 | Ppp2r2a |
| Tmpo | Hyou1 | Fga | Ikzf4 | Ncdn |
| Idh3b | Pttg1ip | Hnrnpul2 | LOC120093742 | Zfp110 |
| Fscn1 | ENSRNOG00000063333 | Prdx2 | Strap | Tbl2 |
| LOC100360413 | RT1-A1n | Kprp | Gpr155 | L2hgdh |
| Rpl34 | Tmprss13 | Rpl23 | Mfn2 | Coro1b |
| Sub1 | Copa | Ifit1 | Ywhaz | Nif3l1 |
| Actb | Ppia | Prkcsh | Ryr1 | Prdx5 |
| Vdac2 | Sae1 | Ddx18 | Atp6v1c1 | Fars2 |
| Ide | Smpdl3b | Actr5 | Ppp3ca | Ddah2 |
| Idh2 | UGT1 | Plod1 | Sympk | Pcmt1 |

| | | | | |
|---|---|---|---|---|
| Poc1b | Cops2 | Cuta | Osbpl9 | Rad23b |
| ENSRNOG00000067128 | Ergic1 | Psma3 | Hp1bp3 | Aar2 |
| Rrm2 | ENSRNOG00000065905 | Dsg1 | B4gat1 | ND3 |
| Wdr5 | Itih3 | Hikeshi | Rpe | Brd2 |
| Snrpd1 | Ndufa10 | Smc2 | ENSRNOG00000069209 | Ywhae |
| Mtpn | Hba-a1 | Prpf4 | C4 | Rab12 |
| Rpl11 | Adrm1 | Coa6 | Pik3r4 | LOC286987 |
| Sgms2 | Serpina3a | Ifit3 | Abcf2 | Nup107 |
| Ssrp1 | Rabggtb | Impa2 | Idh3a | Krt78 |
| Acad10 | Cd82 | Hnrnpl | | Dpp3 |
| Dnaja1 | Nampt | Rpl31 | Ptpa | Actn4 |
| Nub1 | Shmt1 | Qars1 | Ythdf2 | Qdpr |
| Man2a1 | | Snrnp70 | Rab8a | Kidins220 |
| Rpl6 | Mcm6 | Arfgef2 | RT1-A | Crot |
| Uba1 | Timm50 | Ube2d3 | Rtn4 | Acat2 |
| Krt16 | Dhrs4 | Pcyox1 | Hnrnpa0 | Hnrnpf |
| Aldh3a2 | Sec63 | Selenot | Itgav | Sdhb |
| Alb | Oasl | ENSRNOG00000070648 | Fermt3 | Farsa |

| | | | | |
|---|---|---|---|---|
| Ap2m1 | Stambpl1 | S100a16 | Calu | Top1 |
| Atxn10 | Midn | Coq8b | Iars1 | Rpf2 |
| Pafah1b1 | Ilf3 | Poglut2 | LOC100360610 | Cdc16 |
| Dnmt1 | Tor1aip1 | Gusb | Rtcb | Ap1s1 |
| Lgals3bp | Myof | Tmem43 | Esd | Got1 |
| Slc25a11 | RGD1560212 | Bcap31 | Ppia | Mtch2 |
| Cs | Tuba1c | Dhx35 | Ndufa4 | Psme4 |
| Krt72 | Nedd4 | ENSRNOG00000062895 | Gdpd1 | Gchfr |
| ENSRNOG00000068856 | Ctbp2 | Plxnb2 | H4f3 | Rab21 |
| Hadha | Slc25a13 | Gstm2 | Hdlbp | Wdr77 |
| Cyc1 | Blvra | Tfg | Suds3 | Ezr |
| Krt42 | Cops4 | Pnp | Lsm5 | Snx27 |
| Oas1a | ENSRNOG00000070455 | Rpl35 | Anp32b | Slc12a4 |
| Pkp1 | Pgm2 | Gnb2 | Gdi2 | Pak1ip1 |
| Psmb1 | Galm | Flii | Tkt | Slc25a10 |
| Psmd3 | | Strn3 | Snd1 | Kpna6 |
| Ncl | Clcc1 | Dpm1 | Napa | Samhd1 |
| Rbpj | Tns1 | Zdbf2 | Acsl3 | Akr1b1 |

| | | | | |
|---|---|---|---|---|
| Cap1 | Asns | Vps26a | Myh14 | Ddx24 |
| Acadm | Ythdf1 | Sppl2a | Mgst2 | Arcn1 |
| Atox1 | Atp6v1a | Kpna1 | Naga | RGD1564617 |
| Usp7 | Mybbp1a | Hint1 | Gsto1 | Ash2l |
| Ptges3 | Slc6a8 | Hsp90b1 | Ociad1 | Nono |
| Aamp | Rpf1 | Ftsj3 | Rps28-ps3 | Ddx27 |
| Krt76 | Eif2ak4 | Shmt2 | Myg1 | Get3 |
| Dsc1 | Myo1e | Rpap1 | Taf3 | Hdac2 |
| Arhgdia | Washc4 | Skic8 | Dad1 | COX1 |
| Aldh18a1 | Ehd2 | Rtraf | Ubxn8 | Atp5po |
| Ppp1cc | Fth1 | Eif4a3 | Nudt21 | Ermp1 |
| Hnrnpa2b1 | Arf3 | RGD1561333 | Csnk2a1 | Os9 |
| Uap1 | Trappc4 | Ddx39b | Dync1i2 | Naalad2 |
| Serpinc1 | Med23 | Clcnka | Psmd4 | Api5 |
| Fabp5 | Txnrd2 | Tmem165 | Plaa | Mgat2 |
| Cyb5a | Slc35b2 | Xdh | | Dnajc5 |
| Dkc1 | Ndufs3 | Usp47 | Gorasp1 | Lrp1 |
| Hk1 | Mydgf | Kdelr2 | Dnajc2 | Rpl7 |
| Dld | Cert1 | Ddt | Tbcd | Tbc1d15 |
| Cav1 | Selenbp1 | Gstm3 | Tmx2 | Mrps27 |
| Rpl3 | Csnka2ip | ENSRNOG00000069084 | Crnkl1 | Arsa |

| | | | | |
|---|---|---|---|---|
| Elmo1 | Hars1 | Hnrnpab | Manf | Metap1 |
| Park7 | Tf | Trip13 | Iars2 | Msh6 |
| Rps6 | Sacm1l | Ppp4r3b | Foxred1 | Cd81 |
| ENSRNOG00000064820 | Acadsb | Tmem245 | Plbd2 | Uggt1 |
| RGD1564696 | Atad3 | Baz1b | Renbp | Rab9a |
| Eif4a1 | Tarbp1 | Ergic3 | Agl | Cpt1a |
| Tmx1 | Ctps1 | Hnrnpc | Hpcal1 | Cct7 |
| Msn | Cyb5b | RAKi | Ugp2 | Nedd1 |
| Xrn2 | Nasp | Gstp1 | LOC690000 | Mtch1 |
| Tln1 | Lnpk | RGD1306717 | Smarcc2 | Cluh |
| Psmb10 | Ddx17 | Capn1 | Adpgk | Psmc4 |
| Mat2a | Dpep1 | Rad23a | Cdk6 | Sephs1 |
| Fkbp1a | Top2a | Dapk3 | Fbxw11 | Tmem30a |
| Ppif | Pkn3 | Rab2a | Arhgdib | Copg1 |
| Hspa4 | Arsb | Mapk3 | Creld2 | Agap3 |
| Etfa | C1qb | Kpna3 | Ccar1 | Gpt |
| Prpf19 | ENSRNOG00000067516 | Pde2a | Wdr45b | LOC103691761 |
| Lgals9 | Nfasc | Tecr | Acads | Tmem126a |
| Psmb4 | Hsdl1 | Bcat2 | Krt83 | Pa2g4 |
| Prkar2a | Eif4g1 | Enpp5 | Lsm7 | Gorasp2 |

| | | | | |
|---|---|---|---|---|
| Skp1 | Snrpa1 | Scp2 | Ppic | ENSRNOG00000070633 |
| Psma5 | Pzp | Prmt1 | Mrpl48 | Tuba4a |
| Ctsb | Dnajb6 | Kpna4 | Islr | Aga |
| Vcl | Cdk2 | Fads1 | Cct4 | Rars2 |
| Scpep1 | Psmd13 | Pdzd2 | Chgb | Ctsz |
| Psmc3 | RGD1564606 | Septin11 | Snrpf | Psmd11 |
| Igf2 | Nip7 | Rps27l | Numa1 | Sprr2d |
| Insrr | Csk | Ncbp2 | Cstf3 | Slc25a4 |
| Erich2 | Gdi1 | Rpl35a | Mpeg1 | Wdr12 |
| Hnrnpul2 | Psmc2 | Eef2 | LOC100360846 | Slc22a1 |
| Rack1 | Eftud2 | Tor1aip2 | Gcdh | Snrpd3 |
| Prdx2 | Gch1 | Hsd17b11 | LOC100365839 | Tamm41 |
| Kprp | Nomo1 | Ipo9 | Got2 | Blvrb |
| Ifit1 | Nploc4 | Mif | Psmd8 | |
| Prkcsh | | AABR07018058.1 | Epb41l1 | Lamtor2 |
| Psma3 | Eno1-ps1 | Txnl1 | Tacc3 | Leprot |
| Dsg1 | Ddx19b | LOC303448 | Slc27a4 | Sf3b3 |
| Hikeshi | Rps13 | Psme2 | ENSRNOG00 | AABR070172 |

| | | | | |
|---|---|---|---|---|
| | | | 000063418 | 50.1 |
| Coa6 | Arf4 | Eef1e1 | Psma1 | Cul5 |
| Cycs | Mgst1 | Gpr107 | Dsp | Chaf1b |
| Krt15 | Arhgap1 | Cope | Wdfy1 | Psmd12 |
| Hnrnpl | Anxa4 | Akr1b10 | Hnrnph1 | Txn |
| Rpl31 | F2r | Ythdf3 | Adk | LOC10036245<br>3 |
| Snrnp70 | Dnpep | Ube2z | Csnk2b | Slc39a8 |
| Pcyox1 | Atp7a | Sh3bgrl | Wars1 | Hp |
| ENSRNOG00<br>000070648 | Mtr | AABR070608<br>72.1 | Mcfd2 | Ehbp1l1 |
| Aldh9a1 | Mrps5 | Ptgfrn | Fis1 | Chm |
| ENSRNOG00<br>000062895 | Bzw1 | Appl1 | Klhdc4 | |
| Plxnb2 | Bles03 | Cfl2 | Nsf | |
| Tfg | LOC681458 | Rps11 | Etfb | |
| Krt34 | Cavin1 | Cnot1 | Gla | |
| Gnb2 | Sel1l | Cct2 | Prrc1 | |
| Flii | Memo1 | | Pde6d | |
| Cfi | AABR070606<br>10.1 | Hnrnpk | Snrnp40 | |
| Strn3 | Twsg1 | Sdha | Fv1 | |

| | | | |
|---|---|---|---|
| Hsp90b1 | Krt77 | Il6 | Acot7 |
| Shmt2 | Fyb2 | Epb41l5 | Thoc2 |
| Eif4a3 | Rpn2 | Kdm1a | Serinc1 |
| RGD1561333 | Cisd1 | Krt5 | Rpl13a |
| Ddx39b | Alcam | Ighm | Chordc1 |
| Ddt | Ppp4r4 | Snrpd2 | Mat2b |
| ENSRNOG00000069084 | Naglu | Gstm4 | Acy3 |
| Gstp1 | Rrbp1 | Lsm4 | Clic1 |
| Rps27l | Lonp1 | Snupn | Araf |
| Eef2 | Stat1 | Tefm | Surf1 |
| Txnl1 | Acot1 | Gnl1 | Cdk1 |
| Rab6b | Pitpnb | LOC100909916 | Utp18 |
| Sh3bgrl | Gsta4 | Cdc23 | Rfc5 |
| AABR07060872.1 | Eif3a | Rab5if | Phb2 |
| Gnb1 | Atic | Vars1 | Yy1 |
| Ptgfrn | Tbk1 | Akr7a2 | Slc25a5 |
| Hnrnpk | Gmps | Pgam1 | Rpa1 |
| Sdha | Dpcd | Ap2a2 | Stt3a |
| Tuba1b | Hras | | Psmd14 |

| | | | |
|---|---|---|---|
| Pof1b | Map1b | Tgm2 | Dynll2 |
| Krt5 | Caprin1 | Gpatch11 | Hbs1l |
| Gstm4 | Rpl26 | Ndufb5 | Heatr1 |
| Lsm4 | Cyb5r3 | Lpgat1 | Mcat |
| Vars1 | Eif2a | Snrnp200 | Tusc3 |
| Pgam1 | Ahcyl1 | H6pd | Smg5 |
| Tgm2 | Itih4 | ENSRNOG00 000067643 | Nt5c2 |
| Snrnp200 | Chmp4b | Tnpo3 | Npc1 |
| H6pd | Psmd5 | Wipi2 | Prpsap2 |
| Wipi2 | Prdx1l1 | App | Myl12a |
| App | Gpx1 | Anxa6 | Arfgap2 |
| LOC686143 | Nf1 | Tap2 | Cstf1 |
| Rab7a | Ascc3 | LOC686143 | Cltc |
| Dtd1 | Tatdn1 | Cab39 | Fdps |
| Akr1e2 | Col6a3 | Lyl1 | Kif3a |
| Prdx4 | Ctbp1 | Aspa | Ranbp3 |
| Fabp4 | Utp15 | Spag9 | Sec24d |
| Rab5c | Naxe | Rab7a | Mix23 |
| Cpne1 | Dhodh | Dtd1 | Ppp6r3 |
| Itgb1 | Pi4k2a | Sbds | Igf2bp2 |
| Ahcy | Rps17 | Cspg4 | Abhd13 |

| | | | |
|---|---|---|---|
| Prdx6 | Mob4 | Gpd1 | Atp5f1b |
| Fn1 | Alad | Zfr | Pdcd6ip |
| Psmb7 | Col1a1 | Dhrs1 | Psma4 |
| Mapk1 | Dhfr | Actr1a | Acadvl |
| Cse1l | LOC500959 | Tmem115 | Sec31a |
| P4hb | Hnrnpa1 | Ube2d2 | Rps9 |
| Anxa5 | Actr10 | Ctsl | Oat |
| Ndufv1 | Gbe1 | Prdx4 | Crtap |
| Eif3i | Ndufa5-ps4 | Sart3 | Rps24 |
| Ldha | G3bp1 | Bmp2k | Cfap20 |
| Hsp90ab1 | Igtp | COX2 | Exosc3 |
| Sod1 | Trim56 | Fabp4 | Atp6v1h |
| Usp40 | Psmb2 | Rab5c | Clptm1l |
| Vdac1 | Cops3 | Ppwd1 | |
| Ywhah | Rbbp4 | Cpne1 | Sod2 |
| Jakmip2 | Prkag1 | Mical1 | Tram1 |
| Npm1 | Npc2 | Gls | Snx12 |
| Krt17 | Thumpd1 | Vta1 | Cmtr1 |
| Hibadh | Ywhaq | Asap2 | Cox6b1 |
| Eif3l | Tsn | Itgb1 | Atp6v0c |
| Mlec | Tecpr1 | Akap7 | Abhd14b |
| Pdia3 | Slc16a1 | Ahcy | Mpc2 |

| | | | |
|---|---|---|---|
| Pgk1 | Hspa5 | Nit1 | Sqor |
| Eif6 | Wdr83 | Steap1 | Tmed10 |
| Tmed7 | Sec11a | Actl6a | Itga5 |
| Hspe1 | Gnai3 | Prdx6 | Tial1 |
| Wdr82 | Xxylt1 | TAP2C | LOC306079 |
| RGD1559972 | Coq9 | ENSRNOG00 000070918 | Cebpz |
| Arl8b | Tjp1 | Rbm15 | Pcbd1 |
| Eno1-ps20 | Ralb | Fn1 | ENSRNOG00 000066380 |
| Pfkm | Tollip | Rcc1 | Prps2 |
| Gnas | Rabgap1 | Smc4 | Lman2 |
| Hsd17b12 | Eif4g2 | U2af2 | ENSRNOG00 000063840 |
| Ipo7 | Rpl10a | Rock2 | Apeh |
| Tmed2 | Nptn | Insr | Filip1l |
| Tgm3 | Lars1 | Psmb7 | Plpbp |
| Pabpc1 | Igf2r | Nsdhl | Ppp2r1a |
| Emsy | Nadk | Ankfy1 | Sfxn3 |
| C1qc | Pdcd6 | Mapk1 | Nckap1 |
| Flnc | Mcm5 | Pcbp2 | COXII |
| Pfn1 | Nudcd1 | Cse1l | Rae1 |

| | | | |
|---|---|---|---|
| Impdh2 | Rsad2 | P4hb | Npepps |
| Dnajc7 | ENSRNOG00 000064589 | Oasl2 | Dtnb |
| Srm | Dnajb1 | RT1-A2q | Pfas |
| Naca | Chchd4 | Naa25 | Hspb11 |
| Mgst3 | Tmem11 | Zc3h15 | Tspo |
| Ppib | Mapk14 | Anxa5 | Agpat3 |
| Nle1 | Lmnb1 | Brwd1 | Psmd1 |
| Plrg1 | Fasn | Eif2s1 | Ghitm |
| Rpl38 | Sec61a1 | Ndufv1 | Bst2 |
| Rpl4 | Prkag2 | Chchd2l2 | Gemin4 |
| Ahsa1 | Ap2a1 | Fam120a | Gys1 |
| Rcc2 | Vps33a | Sec23b | Psmd2 |
| Pebp1 | Mesd | Eif3i | Hyi |
| Mdh2 | Slc1a5 | Nmd3 | Lyz2 |
| Smurf1 | Sdcbp | LOC10036543 8 | Epb41l2 |
| Hbb | Smarca5 | Ltf | Glrx |
| Ptgr1 | Trim32 | Ldha | ENSRNOG00 000063289 |
| Tpt1 | Cpt2 | Pdgfa | Agfg2 |
| Lamb1 | Psmb9 | Fahd1 | Ogdhl |

| | | | |
|---|---|---|---|
| Hsp90aa1 | Rap1gds1 | Ano6 | Rpl19 |
| LOC108352309 | Dek | Sugt1 | Atp13a1 |
| Gpd2 | Ppa2 | Hsp90ab1 | Pi4ka |
| Psma6 | Cdk14 | Sod1 | Alas2 |
| Pgm1 | Tra2a | Usp40 | Prkaa1 |
| Casp6 | Mcm4 | Vdac1 | Heatr5b |
| Psap | Rps16 | Ywhah | Emc1 |
| Nme1 | Acyp1 | Sec61a2 | Aifm2 |
| Myh9 | Mfsd10 | Hdgf | ENSRNOG00000065564 |
| Ywhag | Myo1c | Arl1 | Pfkl |
| Lmna | Xpo7 | Clic6 | Hspa9 |
| Arg1 | Pfkp | Trap1 | Ube2e3 |
| LOC100362479 | Raly | Far1 | Rer1 |
| Psmb8 | Copg2 | Kng1 | Vps26b |
| Pdia4 | Timm13 | Srsf6 | Tars1 |
| Mdh1 | AABR07021465.2 | Spg7 | Pitpna |
| Rplp0 | Fkbp9 | Wdr48 | Acaca |
| Rps3 | Atp2b1 | Hm13 | Nars1 |

| | | | |
|---|---|---|---|
| Stoml2 | Ephx2 | Prcp | Dlst |
| ENSRNOG00000062927 | Actbl2 | Sord | Septin2 |
| RGD1562416 | Hpn | Cdc42 | Ddx5 |
| Por | Map2k1 | Mcm7 | Pmpca |
| Vat1 | Scly | Npm1 | Ppp1cb |
| Igfbp3 | Anapc2 | Mrpl37 | Olr59 |
| Pkn3 | Prpf8 | Wbp2 | Mapre1 |
| Etf1 | Aars1 | Aacs | Lman1 |
| Vim | Anxa7 | Tbc1d1 | Tmlhe |
| NRP | Ube2m | LOC120097744 | Samm50 |
| Rps2 | Mlkl | Hibadh | Cct3 |
| Aco2 | Rpl30l1 | Nup54 | Get4 |
| Acaa2 | Tnpo1 | Eif3l | Hnrnpa3 |
| RGD1561722 | Ankrd40 | Mlec | Cand1 |
| Xpo1 | Psmd3 | Pgp | Gfer |
| H4c2 | Arpc4 | Pdia3 | Atp2a2 |
| Vcp | Eloc | Acad8 | Tgm1 |
| Vwa5a | Tcp1 | Pgk1 | Rps4x |
| Cp | Rab3d | Abce1 | Luc7l3 |
| Flnb | Garin5b | Carm1 | Map2k2 |

| | | | |
|---|---|---|---|
| Ass1 | Aldh16a1 | Aldoa | Col6a2 |
| Col5a1 | RT1-A | Pdk3 | Adh1 |
| Psmc5 | Spcs3 | Dmxl1 | Chkb |
| Ppt1 | Dhx58 | Eif6 | Rfc3 |
| Rpl30 | Pmm2 | Hnrnpul1 | Anxa1 |
| Casp14 | Trim47 | Tmed7 | Kars1 |
| Thop1 | Glrx3 | Wdr45 | Yipf5 |
| AABR07021573.2 | Stxbp1 | Hspbp1 | Ccdc25 |
| Txnrd1 | Cfl1 | Hspe1 | Pck2 |
| Ssr1 | Slc25a1 | Wdr82 | Rras |
| Vps35 | Uchl5 | RGD1559972 | Calr |
| Krt80 | Lta4h | Arl8b | Nudt16l1 |
| Gstm7 | Hcfc1 | Gmpr2 | Lgalsl |
| Prss1 | Lgals1 | Tmem9b | Rpa2 |
| Acadl | Ankrd50 | Pfkm | Tbl1x |
| Upf1 | Tra2b | Gnas | Bcorl1 |
| Chtop | Xpot | Rps27a | LOC108350287 |
| Rap1a | Akap1 | Hsd17b12 | Rpsa |
| Psat1 | Canx | Lsm12 | Lgals3 |
| H1-5 | Dst | Gc | Slc9a6 |

| | | | |
|---|---|---|---|
| Rpl32 | Hectd1 | Ipo7 | Uqcrfs1 |
| Pdia6 | LOC103690796 | Lancl1 | Plec |
| Eif3c | Cpox | Arpc2 | Ccdc47 |
| Kcnab2 | Aldh1l2 | Arf5 | Cbr2 |
| Eif5a | Tmpo | Iqgap3 | Calm2 |
| ENSRNOG00000062879 | Cmas | Capn2 | Rtca |
| ENSRNOG00000066660 | Idh3b | Elavl1 | Aldh3a1 |
| Lsm3 | Fscn1 | Dock7 | Dtd2 |
| ENSRNOG00000066746 | Cdk5 | Nipbl | Impa1 |
| Snrpd2 | Fmnl2 | Car13 | Glg1 |
| Krt14 | Rfc2 | Ptpn1 | Txndc17 |
| Blmh | RGD1566035 | Tgm3 | Htatsf1 |
| Grn | Gga3 | Eml2 | Rab11b |
| Naa15 | Casp7 | Stbd1 | Faiml |
| LOC100912027 | Dars2 | Atp6v0d1 | Cth |
| Ctrb1 | Pip4k2b | Pabpc1 | Hat1 |
| Acot9 | Cct5 | Tomm22 | Krt19 |

| | | | |
|---|---|---|---|
| Hspa8 | Naxd | Anapc1 | Hcfc2 |
| Frrs1 | Tbcb | Smim7 | Gfpt1 |
| Ssbp1 | Acss2 | Fndc3b | Vapa |
| 0 | Clns1a | Flnc | Rhoa |
| Krt2 | AABR07058366.1 | Tomm70 | Cox4i1 |
| Pepd | Azgp1 | Erp44 | Eif4a2 |
| Wdr1 | Vdac3 | Mpg | Psme1 |
| Oscp1 | Gps1 | Pfn1 | Ppp2r5d |
| LOC679899 | LOC100360413 | Prkar2b | Ptpn11 |
| Sec13 | Rbm39 | Rpap3 | RGD1562404 |
| Ap2b1 | Rela | Impdh2 | Polr1d |
| Capzb | Thoc6 | Srm | Hdac1 |
| Septin8 | Rpl34 | Asah1 | Trappc10 |
| Prps1 | Pus7 | Naca | Coa7 |
| LOC120093742 | Ago2 | Ifit2 | Vps35l |
| Strap | Sars1 | Mrps2 | Mogs |
| Ywhaz | S100a14 | COX3 | Eef1g |
| Rps11 | Nsun2 | Asrgl1 | Pafah1b2 |
| Nccrp1 | Amacr | Ero1a | Fkbp5 |

| | | | |
|---|---|---|---|
| Calm1 | Kif14 | Ppib | Eif3f |
| Ppia | Sub1 | Eprs1 | Casp3 |
| AABR07062466.1 | Gnpnat1 | Vapb | Dync1h1 |
| H4f3 | Actb | Abcf1 | Plch1 |
| AABR07061001.1 | Top2b | Nle1 | Eefsec |
| Anp32b | Lnpep | Plrg1 | Gars1 |
| Gdi2 | Elmod2 | Dhx29 | Cpsf1 |
| Pum3 | Sdhd | Dock4 | AABR07021955.1 |
| Gsto1 | Ints8 | Rpl38 | Elac2 |
| Myg1 | Cwc22 | Rpl4 | Acly |
| Taf3 | Dnm1l | RragB | A2m |
| Dad1 | Mov10 | Eif3e | Nbea |
| Nudt21 | Slfn5 | Gspt1 | Kpnb1 |
| Csnk2a1 | Tab1 | Cyfip1 | Dhx9 |
| Plaa | Vdac2 | Ahsa1 | Niban1 |
| | Ddx1 | Rasa1 | Gpi |
| Manf | Dbr1 | Rcc2 | Glo1 |
| Plbd2 | Mettl7a | St3gal4 | Reep5 |
| Arhgdib | Xpo6 | Impdh1 | Gtpbp4 |

| | | | |
|---|---|---|---|
| Creld2 | Pfdn6 | Pc | ENSRNOG00<br>000063171 |
| Krt83 | Nrdc | Pebp1 | Cpsf3 |
| Tpm3 | Ap1b1 | Osbpl6 | AABR070217<br>36.1 |
| Grxcr2 | Mybl2 | Itga7 | ENSRNOG00<br>000063280 |
| LOC10036084<br>6 | Camk2d | Gmppb | Polr2a |
| LOC10036583<br>9 | Ide | Mcm2 | Copb1 |
| Got2 | Tubg2 | Pkm | Marf1-ps1 |
| Psma1 | Stom | Mdh2 | Tomm40 |
| Dsp | Idh2 | Galnt7 | Psma7 |
| Hnrnph1 | Upp1 | Lpl | Prkacb |
| Csnk2b | Grk2 | G6pdx | Gsdma |
| Aldoa | Akr1b8 | Ddx21 | Hnrnpu |
| Clic1 | Ncstn | Ipo8 | |
| Slc25a5 | LOC499407 | Gmds | Stxbp3 |
| Rpa1 | ENSRNOG00<br>000067128 | Selenof | Psmb3 |
| Stt3a | Mad2l1 | Pop5 | Rtn3 |

| | | | |
|---|---|---|---|
| Vapa | Rrm2 | Ptgr1 | Sp110 |
| Atp5f1b | Nop2 | Hnrnpm | Cat |
| Psma4 | Wdr5 | Tpt1 | Krt8 |
| Rps9 | Snrpd1 | Emc2 | Oas1f |
| Rps24 | Mtpn | Rhot1 | Tctn2 |
| Sod2 | Akr1c1 | Ndc1 | Serbp1 |
| Sqor | Pmpcb | Dnajc3 | Lactb2 |
| LOC306079 | Rrm1 | Lamb1 | Nudc |
| Ppp2r1a | Pak2 | Slc25a40 | Tbxas1 |
| COXII | Dhx37 | Gm2a | Rnf13 |
| Psmd1 | Ssrp1 | Hsp90aa1 | Coro1c |
| Bst2 | Nudcd2 | Rpl28 | Ech1 |
| Psmd2 | Nt5c3a | Fxn | Smu1 |
| Lyz2 | Dis3 | Ddx54 | Cct8 |
| | Rps12 | Gss | Eno3 |
| Emc1 | Fbxo38 | Trim28 | Umps |
| Tars1 | Rac1 | Ppp2r2d | Ncbp1 |
| Dlst | Pwp1 | | Vps36 |
| Septin2 | Ehd4 | Washc5 | Rpl24 |
| Ddx5 | C8h11orf54 | Ptbp1 | Fgg |
| Cct3 | Tapbp | Psma6 | Txndc5 |
| Hnrnpa3 | Ankrd44 | Pafah1b3 | Eif3b |

| | | | |
|---|---|---|---|
| Atp2a2 | Eif2s3 | Prmt5 | Wwox |
| Tgm1 | Cpd | Nmt1 | Kif13b |
| Rps4x | Acad10 | Dnaaf10 | LOC103694176 |
| | Gstt3 | Ttll12 | Otub1 |
| Ighm | Phb1 | Atp5f1e | Hnrnpr |
| Calr | Mrc2 | Cybc1 | Serpinb5 |
| Krt23 | Mrpl3 | Manba | Supt16h |
| Tbl1x | Dnaja1 | Pwp2 | Snrpb2 |
| Uqcrfs1 | Erh | Oas3 | Plg |
| Cbr2 | Lsm2 | Psap | Tbl1xr1 |
| Fth1 | Pls3 | Nme1 | AABR07040855.1 |
| Dlat-ps1 | Polr2g | ENSRNOG00000069813 | Mre11 |
| Glg1 | Tsga10 | Banf1 | Zfp207 |
| Krt19 | Nub1 | Myh9 | Plekhg3 |
| Gfpt1 | Man2a1 | Trappc11 | Ano10 |
| Rhoa | Cers2 | Aco1 | Polr2b |
| Eef1g | Rab14 | Trappc1 | Abhd6 |
| Tkt | Acot13 | Ywhag | Pabpn1 |
| Eif3f | Rpl6 | Utp4 | Dcakd |

| | | | |
|---|---|---|---|
| Dync1h1 | Uba1 | Lmna | Klhl22 |
| A2m | C1qa | Tex29 | Gnaq |
| Kpnb1 | Krt16 | Ilf2 | Helz2 |
| Dhx9 | Aldh3a2 | Brix1 | Cdipt |
| Gpi | Trrap | Plscr1 | ENSRNOG00 000069655 |
| Man2b1 | Alb | Birc6 | LOC690675 |
| Glo1 | Nek7 | Nceh1 | Nsfl1c |
| Psma7 | Nup133 | Spryd4 | Src |
| RGD1559639 | Suclg1 | Arg1 | Myo9b |
| Prkacb | Gcn1 | LOC10036247 9 | Slc25a45 |
| Rnh1 | Rbm8a | Ddx6 | Iqgap1 |
| Hnrnpu | Btf3 | Timm10 | Flna |
| Psmb3 | Rsu1 | Nkrf | Ndufs2 |
| Rtn3 | Spg21 | Upf2 | Ttc39b |
| Cat | Gfm1 | Pdxk | Prep |
| Krt8 | Ap2m1 | Tle1 | Gpd1l |
| Slc25a12 | Aifm1 | Psmb8 | Gstm5 |
| Serbp1 | Ufd1 | Slc25a20 | ENSRNOG00 000065406 |
| Coro1c | Atxn10 | Stn1 | Serpinh1 |

| | | | |
|---|---|---|---|
| Gtf2a2 | Pafah1b1 | Pdia4 | Clpp |
| Sfpq | Them6 | Brox | Ints10 |
| Rpl24 | Dnmt1 | Mdh1 | Cdk4 |
| Txndc5 | Lgals3bp | Xpot | Uchl3 |
| Eif3b | Plcl2 | Rplp0 | Septin7 |
| Hnrnpr | Pla2g15 | Rpl11 | Stat5b |
| ENSRNOG00000070662 | Cul3 | Esyt2 | Dhps |
| Supt16h | Slc25a11 | Col6a1 | Ces1c |
| Jup | Cs | Ttf2 | Akt1 |
| Plg | Krt72 | Rheb | Nup160 |
| Cltc | LOC120102993 | Stoml2 | Nln |
| Nsfl1c | Katnb1 | ENSRNOG00000062927 | Slc25a17 |
| Iqgap1 | B3glct | Ppp5c | Tardbp |
| Flna | Hadha | Polr1c | Tmem106b |
| Cops6 | Itih1 | Rpl17 | Lamc1 |
| Rpl28 | Pgs1 | Pithd1 | Tubb5 |
| Prep | Cyc1 | Gda | Elmo2 |
| | Krt42 | Rab3gap1 | Eif3m |
| Gstm5 | Aldh1a1 | Por | Ube2t |

| | | | |
|---|---|---|---|
| Serpinh1 | Pkp1 | Prdx3 | Csde1 |
| Ppih | Lsm6 | Sri | Rab6a |
| Eno1 | Psmb1 | Ap3d1 | Atraid |
| Tardbp | Vps16 | Spcs2 | Rps3 |
| Lamc1 | Pds5b | ENSRNOG00 000066406 | Arpc5 |
| Tubb5 | Mug1 | Ndufab1 | Nup85 |
| Hspd1 | LOC679539 | Mt-atp6 | Usp5 |
| Csde1 | Ncl | Vat1 | Ift27 |
| Usp5 | Rbpj | Rpl22l1 | Morc3 |
| Ran | Eif3j | Nup93 | Mars1 |
| Rps23 | Timm9 | U2surp | Lrch3 |
| Cyb5r1 | Gnpda1 | Col4a2 | Atp6v0a1 |
| Uqcrc1 | Cap1 | Clcn5 | Anapc5 |
| Il1rap | Cul4b | Igfbp3 | Ran |
| Rpl18 | Aldh6a1 | Yars1 | P2rx4 |
| Ivd | Twf2 | Pigu | Rps23 |
| Rpl7a | Fbxl20 | Isoc1 | Srp54 |
| Krt1 | Idi1 | Huwe1 | Vac14 |
| Fkbp4 | Kyat3 | Cfh | Oxct1 |
| Hprt1 | Aph1a | Aprt | Cyb5r1 |
| Hadh | Tap2 | Dpp9 | Ipo11-ps1 |

| | | | |
|---|---|---|---|
| Rab1a | Acsl1 | Me1 | LOC10036298<br>7 |
| Lsm8 | Ap1m1 | Sf3b2 | Ddx39a |
| Cyp51a1 | Acadm | Prkab1 | Rps11 |
| Lap3 | Atox1 | ENSRNOG00<br>000069797 | Vps29 |
| Etfdh | Hpx | Adam10 | Actc1 |
| Ganab | Aplp2 | Prpf40a | Arpc1b |
| Dhx15 | Mpv17 | Mrpl2 | LOC10036100<br>8 |
| ENSRNOG00<br>000068034 | Lrrk1 | Etf1 | Mrps9 |
| Ddost | Usp7 | Vim | Uqcrc1 |
| Cox6b1 | Nol11 | Dync1li1 | Tm9sf1 |
| Rpia | Ptges3 | ENSRNOG00<br>000064041 | Il1rap |
| Lrrc59 | Krt76 | Bak1 | Tsg101 |
| Cacybp | Strn | Tmc8 | Derl3 |
| Erlin2 | Arhgdia | Rps7-ps23 | Vkorc1 |
| Dstn | Ppidl1 | Fnta | Klhl28 |
| LOC10834969<br>1 | Kntc1 | Svil | Mrpl45 |

| | | | |
|---|---|---|---|
| Ppa1 | Slk | Rps2 | Pdlim5 |
| Krt10 | M6pr | Col19a1 | Nudt5 |
| Rps8 | Sfxn1 | Aco2 | Abhd16a |
| Cd63 | Farsb | Rrp12 | Sppl2b |
| Apmap | Dgat1 | LOC100362927 | Cops5 |
| Ptma | Matr3 | Hip1r | Sucla2 |
| Gar1 | Aldh18a1 | Acaa2 | Rpl18 |
| Dpp7 | Ppp1cc | Hmbs | Kifap3 |
| Flt3lg | Tbcel | Sptlc2 | Ythdc1 |
| Ephx1 | Hmgcs1 | Mrps24 | Hmgcl |
| Pls1 | Slc27a1 | Gnai2 | Setd3 |
| Tubb6 | Lxn | Xpo1 | Ahsg |
| Gpx4 | Hnrnpa2b1 | Plpp3 | Tmem168 |
| Papss1 | Ube3c | Ipo5 | Ivd |
| Coro1b | Parp14 | Pes1 | Nit2 |
| Prdx5 | Sigmar1 | H4c2 | Rasgrp3 |
| Ddah2 | Fblim1 | Vcp | Ptges2 |
| Rad23b | Uap1 | Vwa5a | Noc2l |
| Brd2 | Krt75 | Cp | AABR07018244.2 |
| Ywhae | Dimt1 | Nup188 | Krt1 |

| | | | |
|---|---|---|---|
| Yif1b | Olfm2 | Flnb | Sec24c |
| LOC286987 | Serpinc1 | Csrp1 | Tmem70 |
| Krt78 | Slc25a22 | Ass1 | ENSRNOG00000063876 |
| Dpp3 | Pycr2 | Ccnc | Ap3b1 |
| Actn4 | Sgpl1 | Nemf | ENSRNOG00000069828 |
| Sdhb | Nme2 | Ppp4c | ENSRNOG00000070269 |
| Ap1s1 | Nxt2 | Col5a1 | Dynll1 |
| Got1 | Bckdhb | Psmc5 | Adgre5 |
| Akr1b1 | Man2b2 | Fabp5 | Dhcr7 |
| Arcn1 | Gsr | Ppt1 | Tmx3 |
| RGD1564617 | Exog | Rpl30 | Wdr33 |
| Ddx3 | Zc3hav1 | Focad | Fkbp4 |
| Hnrnpa1-ps20 | Derl1 | Grhpr | Akr1c13 |
| Uggt1 | Cyb5a | Hsd17b4 | Scarb1 |
| Cct7 | Ola1 | Hrg | Mcu |
| Gsn | Ndufb10 | Sec23a | Wdr36 |
| Sprr1a | Aldoart2 | Jtb | Hprt1 |
| Copg1 | Gpaa1 | Bscl2 | Lss |
| B3gat1 | Dennd10 | Fanci | Naa35 |

| | | | |
|---|---|---|---|
| Gpt | Trarg1 | Thop1 | Cops8 |
| Kxd1 | Ap3s1 | Dnajc10 | Gnpda2 |
| Tuba4a | Ddx3x | RGD1564696 | Bub3 |
| Ctsz | Dkc1 | Txnrd1 | Htt |
| Sprr2d | Glb1 | Ssr1 | Hadh |
| Slc25a4 | Ctsd | Srpra | Krt90 |
| Wdr12 | Dhx30 | Psmd6 | Capns1 |
| Snrpd3 | Hk1 | Rnf213 | Rps15a |
| Sf3b3 | Pycr3 | Vps35 | Rpn1 |
| Anxa3 | Apip | Mrpl16 | |
| Sh3bgrl3 | Dld | Paics | |
| Txn | Nup37 | Pdhb | |
| Ndufv2 | Gbp7 | Rptor | |
| Pcbd2 | Kdm2a | Gstm7 | |

**Supplementary Table 2. List of upstream miRNAs of TRPV1 predicted by Targetscan**

| miRNA | Position in the UTR |
|---|---|
| **Conserved sites** | |
| rno-miR-142-5p | 206-212 |
| **Poorly conserved sites** | |
| rno-miR-3584-3p | 21-27 |
| rno-miR-186-3p | 30-36 |
| rno-miR-23b-5p | 49-55 |
| rno-miR-133a-5p | 52-58 |
| rno-miR-138-5p | 52-58 |
| rno-miR-3575 | 53-59 |
| rno-miR-370-3p | 54-60 |
| rno-miR-652-5p | 58-65 |
| rno-miR-488-5p | 69-75 |
| rno-miR-711 | 74-80 |
| rno-miR-759 | 79-85 |
| rno-miR-206-3p | 98-104 |
| rno-miR-1-3p | 98-104 |
| rno-miR-1b | 98-104 |
| rno-miR-761 | 120-126 |
| rno-miR-214-3p | 120-126 |

| | |
|---|---|
| rno-miR-383-3p | 122-128 |
| rno-miR-188-3p | 125-132 |
| rno-miR-1306-5p | 136-142 |
| rno-miR-362-3p | 139-145 |
| rno-miR-329-3p | 139-145 |
| rno-miR-188-3p | 143-150 |
| rno-miR-6216 | 157-163 |
| rno-miR-376c-5p | 161-167 |
| rno-miR-376b-5p | 161-167 |
| rno-miR-92a-1-5p | 164-170 |
| rno-miR-33-3p | 179-185 |
| rno-miR-742-3p | 186-192 |
| rno-miR-488-3p | 189-195 |
| rno-miR-3064-3p | 226-232 |
| rno-miR-218a-1-3p | 247-253 |
| rno-miR-322-3p | 247-253 |
| rno-miR-3542 | 253-259 |
| rno-miR-484 | 254-260 |
| rno-miR-6334 | 254-260 |
| rno-miR-344a-5p | 254-260 |
| rno-miR-3578 | 264-270 |
| rno-miR-216a-5p | 280-287 |

| | |
|---|---|
| rno-miR-216b-5p | 281-287 |
| rno-miR-1912-5p | 287-293 |
| rno-miR-32-3p | 293-299 |
| rno-miR-291a-5p | 327-334 |
| rno-miR-29b-1-5p | 332-339 |
| rno-miR-338-3p | 339-346 |
| rno-miR-539-5p | 349-355 |
| rno-miR-877 | 354-360 |
| rno-miR-421-3p | 365-371 |
| rno-miR-148a-5p | 389-395 |
| rno-miR-148b-5p | 389-395 |
| rno-miR-3064-3p | 414-420 |
| rno-miR-193b-5p | 418-424 |
| rno-miR-326-3p | 421-428 |
| rno-miR-330-5p | 421-428 |
| rno-miR-320-5p | 425-431 |
| rno-miR-702-3p | 430-436 |
| rno-miR-199a-5p | 438-444 |
| rno-miR-5132-3p | 470-476 |
| rno-miR-547-5p | 491-497 |
| rno-miR-224-5p | 494-500 |
| rno-miR-598-3p | 508-514 |

| | |
|---|---|
| rno-miR-1224 | 523-529 |
| rno-miR-3569 | 523-529 |
| rno-miR-758-3p | 527-533 |
| rno-miR-127-5p | 540-546 |
| rno-miR-3120 | 547-553 |
| rno-miR-21-3p | 548-554 |
| rno-miR-20a-3p | 563-570 |
| rno-miR-760-3p | 567-574 |
| rno-miR-135b-5p | 570-576 |
| rno-miR-135a-5p | 570-576 |
| rno-miR-3084d | 588-595 |
| rno-miR-3084a-3p | 588-595 |
| rno-miR-3084b-3p | 588-595 |
| rno-miR-544-3p | 590-596 |
| rno-miR-488-3p | 598-604 |
| rno-miR-3065-5p | 615-621 |
| rno-miR-7a-1-3p | 615-621 |
| rno-miR-759 | 639-645 |
| rno-miR-217-5p | 646-652 |
| rno-miR-6321 | 646-652 |
| rno-miR-6324 | 662-668 |
| rno-miR-21-3p | 667-674 |

| | |
|---|---|
| rno-miR-183-3p | 680-687 |
| rno-miR-433-3p | 685-691 |
| rno-miR-664-3p | 687-693 |
| rno-miR-376c-3p | 696-702 |
| rno-miR-741-5p | 696-702 |
| rno-miR-1-5p | 700-706 |

**Supplementary Table 3. List of upstream miRNAs of CDK5 predicted by Targetscan**

| miRNA | Position in the UTR |
|---|---|
| **Conserved sites** | |
| rno-miR-142-5p | 154-160 |
| **Poorly conserved sites** | |
| rno-miR-874-5p | 25-31 |
| rno-miR-344g | 29-35 |
| rno-miR-344b-5p | 29-35 |
| rno-miR-370-5p | 32-38 |
| rno-miR-1193-3p | 32-38 |
| rno-miR-879-5p | 42-48 |
| rno-miR-532-3p | 61-68 |
| rno-miR-150-5p | 62-68 |
| rno-miR-324-5p | 78-84 |
| rno-miR-201-5p | 84-90 |
| rno-miR-880-5p | 84-90 |
| rno-miR-133a-5p | 89-95 |
| rno-miR-133c | 90-96 |
| rno-miR-296-5p | 102-108 |
| rno-miR-664-1-5p | 106-112 |
| rno-miR-664-2-5p | 106-112 |

| | |
|---|---|
| rno-miR-1956-3p | 106-112 |
| rno-miR-3085 | 107-113 |
| rno-miR-331-3p | 110-116 |
| rno-miR-1843a-5p | 145-151 |
| rno-miR-3551-3p | 163-169 |
| rno-miR-32-3p | 170-176 |
| rno-miR-3564 | 182-188 |

**Supplementary Table 4. Primers for RT-qPCR analysis**

| Gene | Sequence (5′ – 3′) |
|---|---|
| *TRPV1* | Forward: CAGCGAGTTCAAAGACCCAGAGAC |
| | Reverse: GGAGCAGAGCGATGGTGTCATTC |
| *CDK5* | Forward: CTGCTAGGGACACCGACTGAGG |
| | Reverse: GCATTGAGTTTGGGCACGACATTC |
| *miR-142-5P* | Forward: CCGTGCGCATAAAGTAGAAAGCACTAC |
| *pri-miR-142* | Forward: GAACGCACTGATTTCGACCA |
| | Reverse: GCTAACCAGCGTTTTCGTTC |
| *pre-miR-142* | Forward: TGGAGCAGGAGTCAGGAGG |
| | Reverse: GCCGAGGAAGATGGTGG |
| *METTL14* | Forward: CCGTGAAGCGAAGCACAGATG |
| | Reverse: TTCTATGTTGCCAATCTCAGGTTCC |
| *METTL3* | Forward: CTCTCGTAACCTATGCTGACCACTC |
| | Reverse: CCACTGTAGTCAAGTCCTGCTCTG |
| *WTAP* | Reverse: GAACATCCTTGTCATGCGGCTAG |
| | Reverse: CGGCTGCTGAACTTGCTTGAG |

**Supplementary Table 5. List of Antibodies.**

| Antibody | Reactivity | Company | Product number |
|---|---|---|---|
| TRPV1 | Human Mouse Rat | Abmart | M047783 |
| TRPV1 | Human Mouse Rat | Affinity | DF10320 |
| CDK5 | Human Mouse Rat | Abmart | T55409 |
| IgG | All | Abmart | B30011 |
| β -Tubulin | Human Mouse Rat, etc | Abmart | R20005 |
| CGRP | Human Mouse Ra | Affinity | DF7386 |
| Phospho-Serine/Thr | Human Mouse Rat, etc | Abmart | T91067 |
| β -Actin | Human Mouse Rat, etc | Abmart | P30002 |
| $Na^{+}/K^{+}$ATPase | Human Mouse Rat, etc | Abmart | T55159 |
| c-Myc Tag | Human Mouse Rat, etc | Abmart | T55150 |
| Flag Tag | Human Mouse Rat, etc | Abmart | M20008 |
| GST-Tag | All | Abmart | M20007 |
| METTL14 | Human Mouse Rat | Abmart | PC17608 |
| F-Actin | Human Mouse Rat | Abmart | PC1571S |

**Supplementary Table 6. List of Plasmid.**

| Gene | Sequence |
|---|---|
| CDK5 | ATGCAGAA……GTCCCCCG |
| TRPV1 | ATGGAACA……GGGAGAAA |
| TRPV1(406T>A) | ATGGAACA……GGGAGAAA |
| TRPV1(1-390aa) | ATGGAACA……CCTGTTAA |
| TRPV1(391-839aa) | ATGGAAAA……AGAAATAA |

## Supplementary Figure 1. GO and KEGG

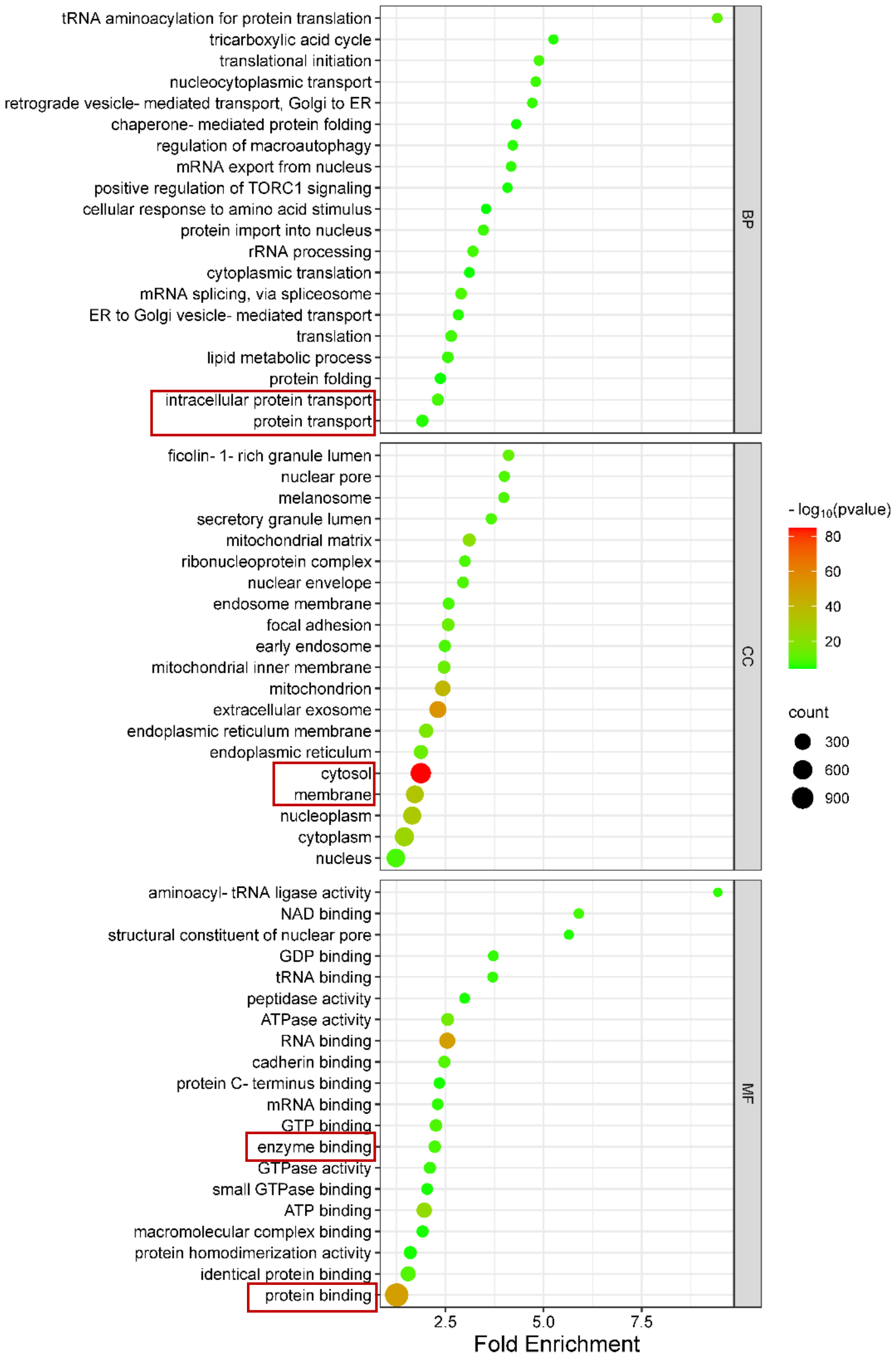

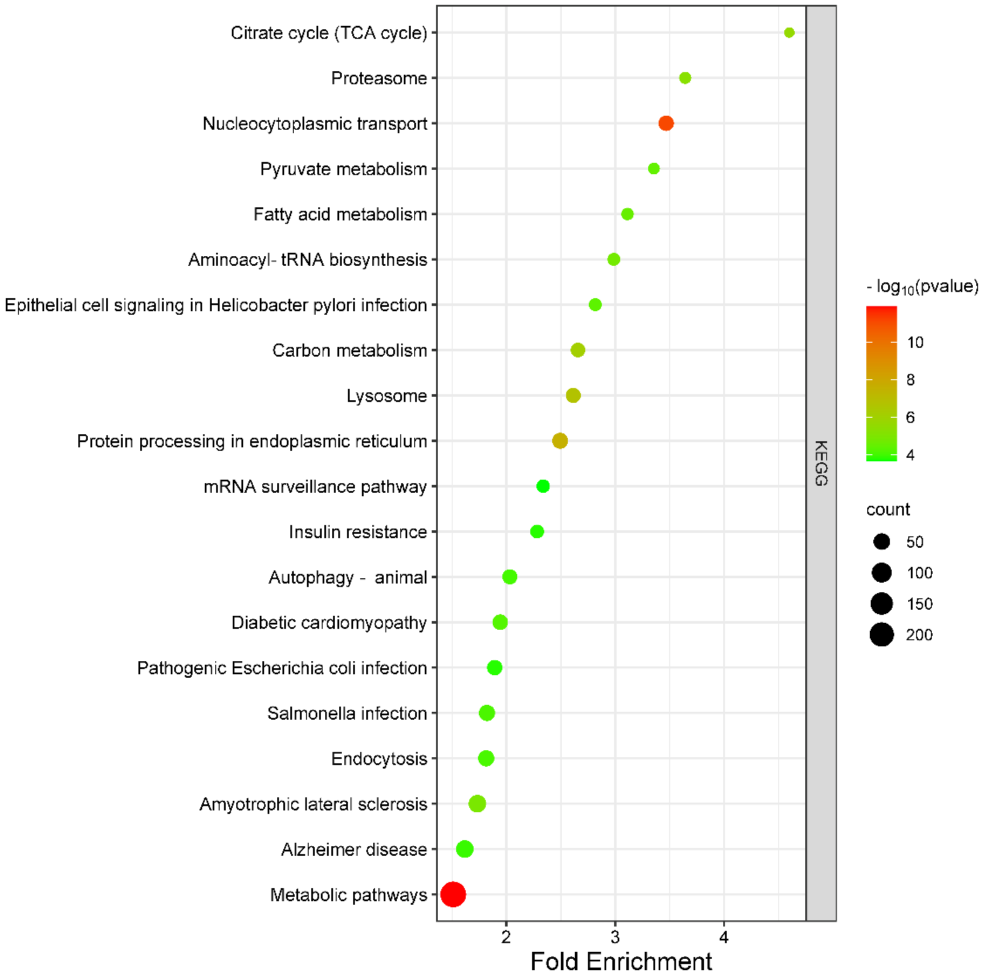


**Fig. S1** The protein sets interacting with TRPV1 were analyzed by GO and KEGG

## Supplementary Figure 2. Efficiency of mimics and inhibitor

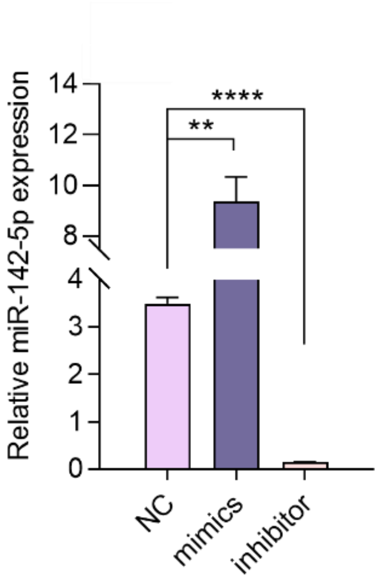


**Fig. S2** Effect of transfection with miR-142-5p mimics or inhibitors on the expression of miR-142-5p in DRG neurons.

**Supplementary Figure 3. Expressions of three common m6A methyltransferases**

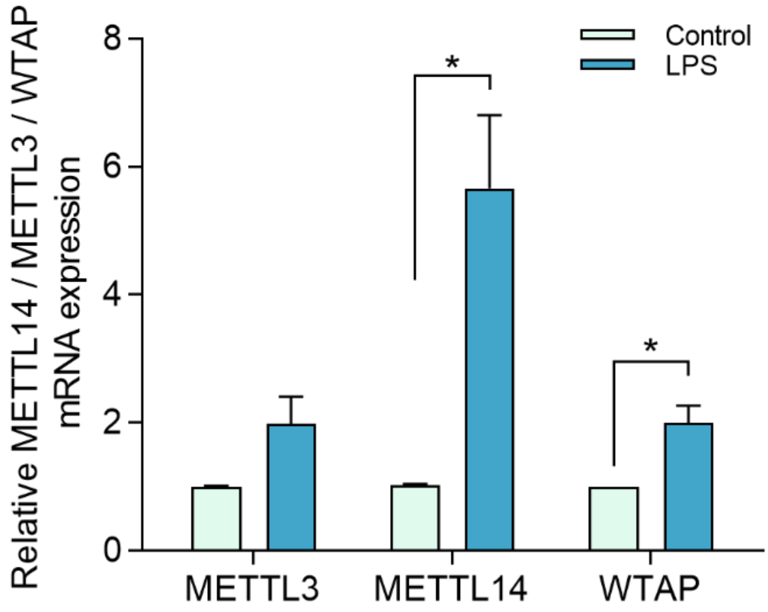


**Fig. S3** The relative mRNA expression levels of METTL3, METTL14, and WTAP.

**Supplementary Figure 4. Paw withdrawal mechanical threshold and paw withdrawal thermal latency in rats after intrathecal injection of agomir**

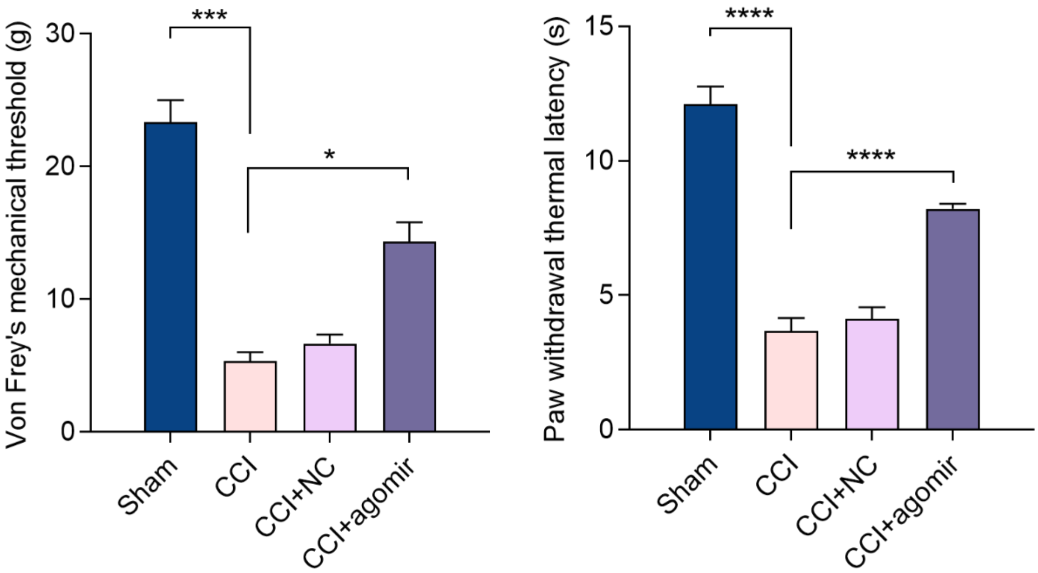


**Fig. S4** The PWMT and PWTL after intrathecal injection of agomir following CCI modeling.
